# Scaffold repositioning of spiro-acridine derivatives as fungi chitinase inhibitor by target fishing and in vitro studies

**DOI:** 10.1038/s41598-023-33279-9

**Published:** 2023-05-05

**Authors:** Jéssika de Oliveira Viana, Eden Silva e Souza, Nicolau Sbaraini, Marilene Henning Vainstein, Joilly Nilce Santana Gomes, Ricardo Olímpio de Moura, Euzébio Guimarães Barbosa

**Affiliations:** 1 Post-Graduate Program in Bioinformatics, Bioinformatics Multidisciplinary Environment, Federal University of Rio Grande do Norte, Natal, Brazil; 2 School of Biomolecular and Biomedical Science & BiOrbic-Bioeconomy Research Center, University College Dublin, Dublin, Ireland; 3 Biotechnology Center, Postgraduate Program in Cellular and Molecular Biology, Federal University of Rio Grande do Sul, Porto Alegre, Brazil; 4 Department of Biological Sciences, State University of Paraíba, Campina Grande, Brazil; 5 Post-Graduate Program in Pharmaceutical Sciences, Faculty of Pharmacy, Federal University of Rio Grande do Norte, Natal, Brazil

**Keywords:** Target identification, Virtual screening, Structure-based drug design

## Abstract

The concept of “one target, one drug, one disease” is not always true, as compounds with previously described therapeutic applications can be useful to treat other maladies. For example, acridine derivatives have several potential therapeutic applications. In this way, identifying new potential targets for available drugs is crucial for the rational management of diseases. Computational methodologies are interesting tools in this field, as they use rational and direct methods. Thus, this study focused on identifying other rational targets for acridine derivatives by employing inverse virtual screening (IVS). This analysis revealed that chitinase enzymes can be potential targets for these compounds. Subsequently, we coupled molecular docking consensus analysis to screen the best chitinase inhibitor among acridine derivatives. We observed that 3 compounds displayed potential enhanced activity as fungal chitinase inhibitors, showing that compound 5 is the most active molecule, with an IC_50_ of 0.6 ng/µL. In addition, this compound demonstrated a good interaction with the active site of chitinases from *Aspergillus fumigatus* and *Trichoderma harzianum*. Additionally, molecular dynamics and free energy demonstrated complex stability for compound 5. Therefore, this study recommends IVS as a powerful tool for drug development. The potential applications are highlighted as this is the first report of spiro-acridine derivatives acting as chitinase inhibitors that can be potentially used as antifungal and antibacterial candidates.

## Introduction

Fungal pathogens are responsible for 13 million infections and 1.5 million deaths annually^1^. Although these numbers are alarming, the severity of fungal infections can vary from asymptomatic to systemic life-threatening diseases. The opportunistic pathogens of the *Aspergillus* genus have emerged as the most frequent cause of fungal diseases^1^. For aspergillosis, triazole drugs are commonly employed as the first line of treatment in clinical therapy. However, these compounds can cause several adverse clinical effects, such as nausea, vomiting, neurotoxicity, and kidney damage^2^. Furthermore, the number of resistant strains has increased at an alarming rate in the past decades^3^. The number of alternative drugs for aspergillosis treatment is scarce and these compounds can be very toxic to already debilitated inpatients^4^. Therefore, new and more efficient alternative antifungal treatments are urgently required.

Chitin is composed of d-glucosamine and N-acetylglucosamine monomers^5^. After cellulose, chitin is the most abundant natural polymer, acting mostly as a structural component in crustacean shells, cell wall of fungi, exoskeletons, and cephalopod beaks, among others^6^. Chitinase (EC 3.2.1.14) are glycoside hydrolase (GH) superfamily members. These enzymes are responsible for the hydrolysis of β-1,4 glycosidic bonds in chitin polymers^7,8^. Among the several GH families, chitinases can be mostly found in the families of GH18 and GH19, with almost all fungi chitinases belonging to the GH18 family^9^. In these organisms chitinases can be employed for cell wall remodeling and as virulence factors^10^.

Given the relevance of these enzymes in fungal cell wall remodeling, chitinases can be attractive targets for the development of new antifungal drugs. The knockout of chitinase genes in fungal pathogens impacts the cell wall division^11–13^. Additionally, chitinase inhibitors (e.g. methylxanthines) can drastically affect the hyphal morphology of fungal pathogens, such as *A. fumigatus*. The mode of action shows that these compounds act as competitive inhibitors against the chitinase of the fungal family 18. In this action, π–π stacking interactions are formed with tryptophans conserved in the active site of this protein, mimicking the action of the intermediate analogues of the reaction, which include Allosamidin (a pseudotrisaccharide), Argifin, and Argadin (a peptide-based product)^14^. Thus, chitinase inhibitors can present a significant protective effect in invasive pulmonary aspergillosis^15,16^.

Among thoroughly explored therapeutic compounds are the derivatives of acridine. These derivatives display good potential against a variety of therapeutic targets, as these compounds show antiparasitic, antiviral, antibacterial, and antitumor activities^17–19^. These effects are usually linked to the inhibition of DNA replication, since these compounds can inhibit type I and II topoisomerase, and telomerases^20^. However, the potential activity of acridine derivatives against other potential targets still needs to be addressed. The term “one target, one drug, one disease” has been the dominant concept in traditional drug discovery. Nonetheless, this paradigm implies that a drug is designed to modulate a single target for specific diseases, when in reality it is known that this is not always true^21^.

The identification of potential targets for a known bioactive compound is fundamental for drug design and development. Experimental methods aiming to identify potential targets with reliable accuracy include protein isolation and subsequent mass spectrometry analysis, as well as mRNA expression-based approaches^22^. However, these strategies are costly and time-consuming. In this way, computational methods based on drug design have been widely used^23^. Such tools have been explored and experimentally validated in a wide variety of applications^13,24^. Currently, Computer-Aided Drug Design (CADD) methodologies are divided into two main classifications: Structure-Based Drug Design (SBDD) and Ligand-Based Drug Design (LBDD). LBDD is based on the design of small ligands with known activities, extracting information about their molecular characteristics^23,25^. On the other hand, SBDD is applied when 3D structural information on the molecular target is used to simulate intermolecular interactions with another molecule. Many examples, such as inverse virtual screening, molecular docking, molecular dynamic simulations, are related^25,26^.

Recently, our research group identified antileishmanial and antitumoral activity for acridine and spiro-acridine derivatives^18,27–29^, which have been shown to be potential drug candidates. However, the few therapeutic applications reported in the literature make us wonder about the best activity performance of these compounds against a target. To address new potential targets of acridines, an inverse virtual screening (IVS) based on the receptor was performed, which pointed to fungal chitinases as ideal targets. Thus, several in silico assays (such as molecular docking and molecular dynamic), as well as in vitro assays were employed to evaluate the mechanism of action and potential inhibitory activity of selected acridine derivatives on fungal chitinases.

## Results

### Target fishing applied to acridine derivatives

The top 550 (highest docking scores) inverse virtual screening results were analyzed by human inspection. The list of the most probable targets for the investigated acridine derivatives are presented in Supplementary Table S1. We found 10 chitinase targets with scores between − 10.6 to − 9.2 kcal/mol. The best targets for the investigated small compounds were chitinases from *A. fumigatus* (*Af*ChiB) (− 10.6 kcal/mol) and from *Serratia marcescens*. The latter presented 7 different entries ranging from − 10.4 to − 9.2 kcal/mol. Other species were discovered, such as the moth *Ostrinia furnacalis* and the bacterium *Vibrio harveyi*, with − 9.4 kcal/mol and − 9.3 kcal/mol, respectively.

**Figure 1:**
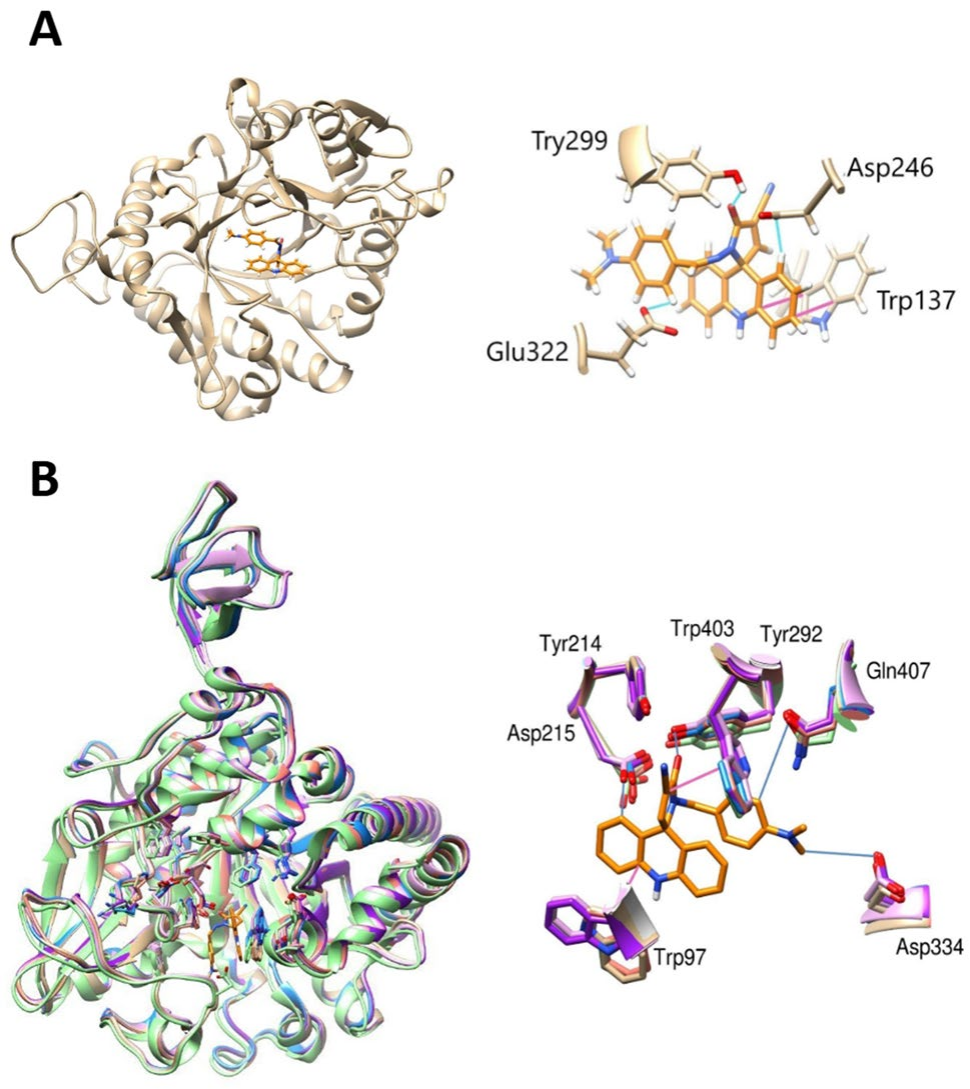
Structural comparison of the results obtained by the IVS. The orange molecule represents an acridine derivative, the blue line represents the H-bond and the pink lines represent π–π interactions. (**A**) *A. fumigatus* is colored gold (**B**) *S. marcescens*, where the green molecule is PDB ID 4Z2G, 3WD1 is colored lilac, 4Z2L is colored gold, 3WD2 is colored light blue, 4Z2J is colored pink, 4Z2K is colored purple and 4Z2I is colored blue.

For *A. fumigatus* the position of the acridine ring overlaps with the aromatic ring of the PDB inhibitor (caffeine) (Fig. 1). The amino acid Tyr29 showed π–π interactions with acridine fragments, since Gly322, Asp246 and Tyr299 showed an H-bond with the ligand. This conformation may indicate a possible activity of functional mimicry^14^. In regard to chitinases of *S. Marcescens,* we observed the 3D overlay of structures, which present the same protein structure. Also, the acridine derivative presents similar interactions with PDB inhibitors^14,30,31^. The π–π interactions were observed between Trp97 and Trp403 with acridine fragments. The H-bond may indicate greater stability of the compound at the site, indicated by Asp215, Tyr292, Gln407, and Asp334.

Despite the structural differences, the active site for these enzymes is conserved, which helps to explain the score values for the IVS. In addition to the DXDXE domain in the active site (Fig. S1), the presence of a nonpolar region with aromatic amino acids, and the presence of polar amino acids, stand out. This feature assists in the positioning of the acridine compound, promoting the intercalations of aromatic rings and H-bond formation. Such characteristics have been shown to be crucial to ensure the positioning of the derivative in the active site.

### Chitinase alignment structure shows similarities in domains

To evaluate the activity of the acridine derivatives for the chitinase isoforms of *A. fumigatus* (*Af*ChiB), alignment was created based on the chitinase structure of PDB ID 2A3B. Protein alignment shows 61% identity and 90% coverage of *T. harzianum* (Chit33 and Chit42) with *A. fumigatus* (Fig. S1). The active site and the presence of the DXDXE domain are similar among the species. It is observed that *Af*ChiB shows greater identity with Chitinase 42 from *T. harzianum* (Chit42), with the same active site (Fig. S2).

### Molecular docking simulation and consensus analysis

In addition, molecular docking studies were applied to chitinase isoforms. Results indicate that acridine derivatives interact with amino acid residues in the active site (Tables S2, S3). Using AutoDockTools v. 1.5.7, the Root Mean Standard Deviation (RMSD) of the redocking of *A. fumigatus* inhibitor was calculated, with an RMSD of 1 Å (Fig. S3). The RMSD calculation helps to compare the position of a crystallized ligand with the position of a simulated ligand in the active site of the same protein. With this analysis, known as redocking, it is possible to indicate that the simulation presented a conformation similar to that of the crystallizing ligand, which could serve as a validation of the docking procedure. A value between 0 and 2 Å could validate the docking simulation^32^.

All acridine derivatives interact with the DXDXE motif, which is involved in the catalysis and loss of catalytic activity of chitinase^8^. The acridine derivatives showed better energy value with chitinases isoforms from *A. fumigatus* and *T. harzianum*, when compared to the caffeine compound. Among acridine derivative classes, it is possible to note that the spiro-acridine derivatives showed a stronger interaction when compared to the thiophene-acridine and acridine-thiosemicarbazides. To perform a consensus analysis between molecular docking, it was observed that compounds 5, 7 and 9 were shown to be the top hit compounds. These compounds were selected for in vitro activity in the *T. harzianum* enzyme, the model species for chitinase assay.

As already highlighted, the DXDXE domain, responsible for the catalytic activity, is present in the studied isoforms. It is observed that the compounds interacted well with this domain, demonstrated by the Asp and Glu residues with H-bond. In regard to *A. fumigatus*, it was observed that the active site has a more hydrophobic and polar region. The score values showed compound 5 was more potent (− 10.9 kcal/mol), followed by compound 9 (− 10.6 kcal/mol), with compound 7 having the lowest binding (− 10.1 kcal/mol). When comparing the score values with the PDB inhibitor, caffeine (− 6.3 kcal/mol), it is observed that our acridine derivatives show stronger interactions with the active site (Table S2).

**Figure 2:**
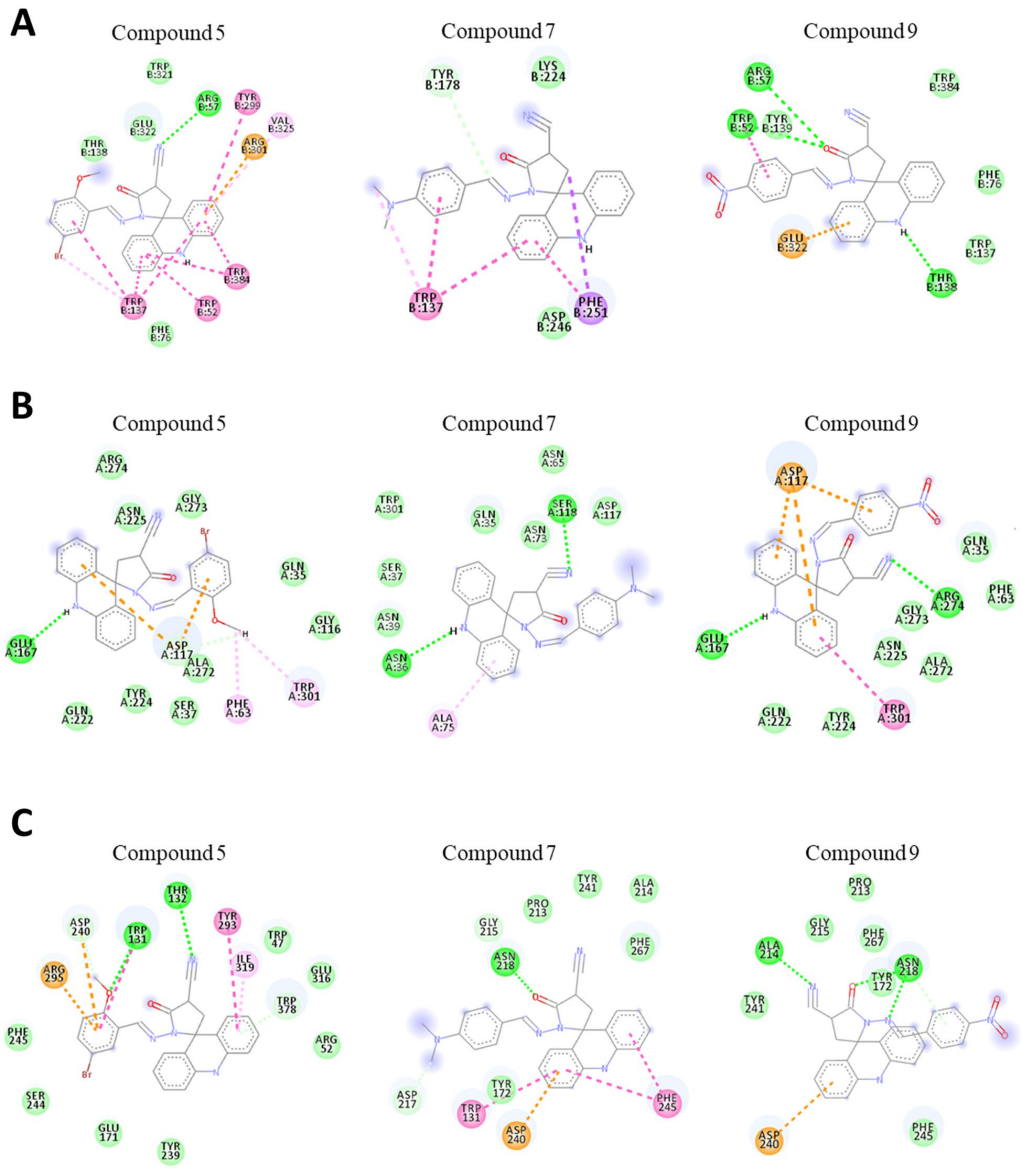
Molecular docking simulation of compounds 5, 7 and 9 in chitinase isoforms for the species *A. fumigatus* and *T. harzianum*. The green lines representing the H-bond, pink as π–π, light pink as π-alkyl and orange as π-charge between amino acid residues and compounds. (**A**) Interactions of compounds with *A. fumigatus* chitinase. (**B**) Interactions of compounds with *T. harzianum* Chit33 isoform. (**C**) Interactions of compounds with *T. harzianum* Chit42 isoform.

Compounds 5 and 9 have similar conformations in the active site. Thus, the formation of stacked π–π interactions with the acridine fragment, exchanged with Trp52 and Trp138, stands out. On the other hand, there is a more polar region at the site, composed of the amino acid residues Arg57 and Thr138. This region, where the protein domain is inserted, favored the presence of the H-bond with the aromatic portions of the acridine derivatives (Fig. 2A). However, compound 7 presents a different conformation in the active site, being highlighted by the H-bond of Tyr178 with the acridine ring. The π–π stacked bonds were observed for the aromatic portions, highlighted by the residues Phe251 and Trp137 (Fig. 2A).

The *T. harzianum* species have two chitinases, known as Chit33 and Chit42^33^. In regard to *T. harzianum* chitinase, it is observed that the acridine portion interacts positively with the active site region of two isoforms. There is a difference between the active site of the isoforms: for Chit33 the site presents more polar amino acids, while Chit42 favors the presence of aromatic and polar amino acids. For both isoforms, the compounds present interactions with the catalytic domain of chitinase. Due to the structure of acridine derivatives presenting a high prevalence of aromatic portions and H-bond, the score values were higher for Chit42, indicating greater interaction in this complex (Fig. 2B).

For the Chit33 isoform compound 5 was more potent (− 7.8 kcal/mol), followed by compound 9 (− 7.7 kcal/mol), and compound 7 having the lowest binding (− 7.3 kcal/mol) (Table S3). Compounds 5 and 9 present similar conformation and interaction, such as an H-bond with the amino acid residues Glu167, and Asp117 to acridine and pyrrolidine groups. Also, Trp301 showed the π–π bonds with the acridine fragment, being crucial for the interaction of the complex (Fig. 2B). Compound 7 was divergently positioned at the active site of the chitinase 33 isoform (Fig. 2B). The acridine fragment performed an H-bond with Asn36 and Ser118. The benzene ring, on the other hand, formed π–π bonds with the residue Ala75, indicating that this group favors the interactions of the complex.

The simulation showed compound 5 as the most potent (− 9.8 kcal/mol) for Chit42, followed by compound 9 (− 9.3 kcal/mol), and compound 7 had the lowest binding (− 9.5 kcal/mol) (Table S3). In the active site there is a high occurrence of aromatic amino acids that favored the presence of π–π interactions. Additionally, the active site integrates more polar regions with inhibitors, being exchanged by water molecules, stabilizing the structure in this region (Fig. 2C).

For the Chit42, compounds 5 and 9 showed similar positioning in the active site. Interactions were mostly driven by the presence of carboxyl and acridine groups when forming an H-bond with Asp240. The compound 5 also aligned the acridine group with the amino acid residues Trp378 and Tyr293, forming π–π interactions and a bromobenzene group with Trp131 and Arg295. On the other hand, the compound 9 also demonstrated high stability in the active site, in which the residues Ala214 and Asn218 stand out (Fig. 2C).

For compound 7, it is observed that the presence of the dimethylaniline group influenced the positioning of the compound, failing to create an H-bond with residues Asn218 and Tyr172, as observed for the other compounds. Instead, the presence of acridine interacted with the Phe245 and Trp131 residues, indicating that its presence is essential to allow π-stacked interactions to happen. It was also observed that the acridine core interacted with π-stacked bonds with Asp240 residues (Fig. 2C).

### Inhibition of chitinolytic activity

**Figure 3:**
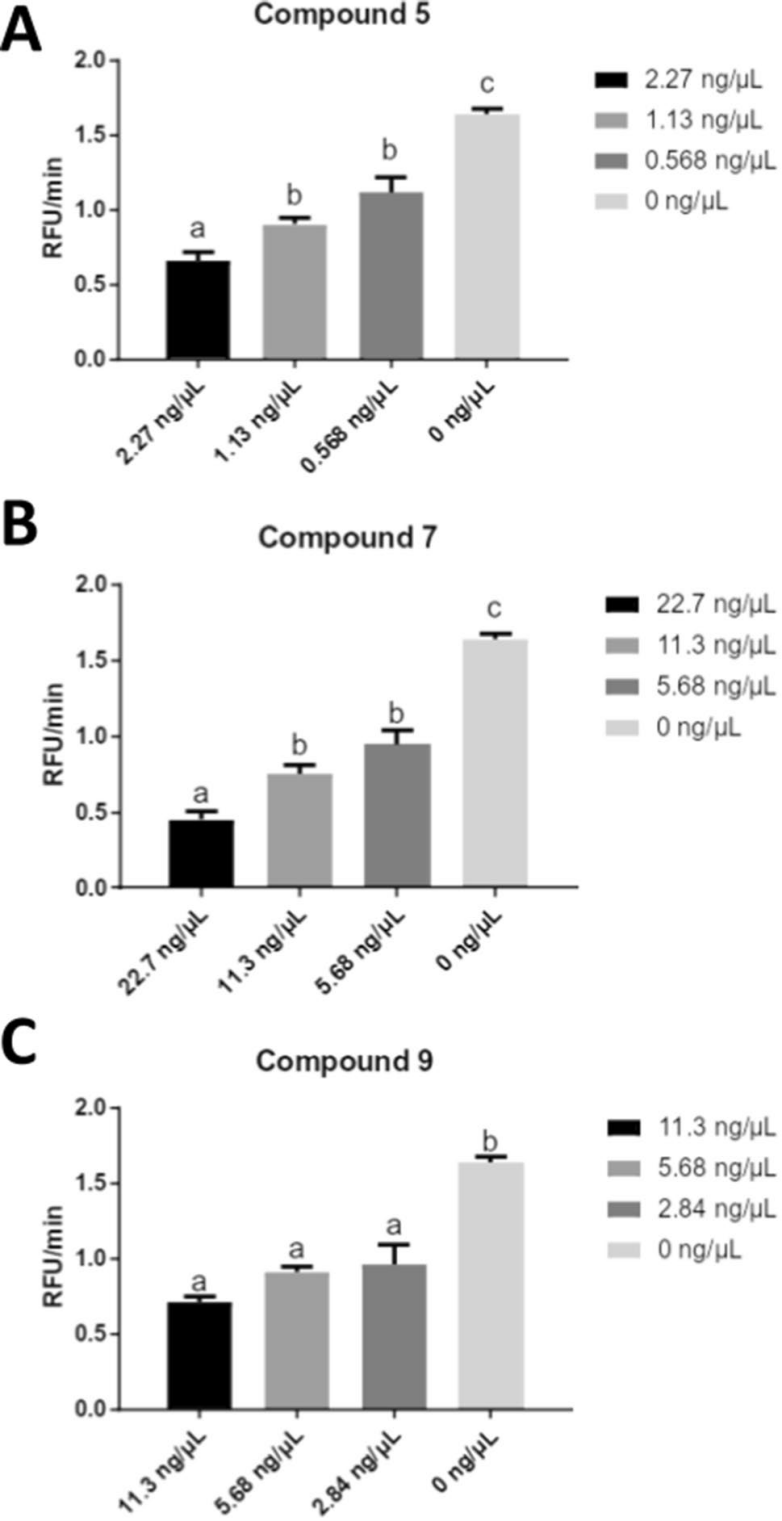
Chitinolytic activity assay. Inhibition of chitinolytic activity by: (**A**) compound 5 (**B**) compound 7, and (**C**) compound 9. Statistical significance is indicated as letters above the bars.

Given the in silico results, we set out to evaluate the potential inhibitory activity of the acridine derivatives on a lysing enzymes mixture obtained from *T. harzianum*. All tested compounds inhibited chitinolytic activity in a dose-dependent manner (Fig. 3). It is noteworthy that compound 5 exhibited remarkable inhibition of chitinolytic activity. At the highest concentration tested (2.27 ng/µL) (Fig. 3A), compound 5 exhibited a 2.4-fold reduction in activity. Furthermore, the observed IC_50_ for this compound was 0.6 ng/µL (Fig. S4). Similarly, at the highest concentration tested of 22.7 ng/µL (Fig. 3B), compound 7 showed a 3.6-fold reduction in chitinolytic activity, when compared to the control without the compound, and an IC_50_ of 5.7 ng/µL (Fig. S4). Finally, a 2.2-fold reduction in chitinolytic activity was observed when 11.3 ng/µL of compound 9 was used (Fig. 3C), with an IC_50_ of 1.8 ng/µL (Fig. S4). These results indicate the potential of these compounds, especially 5, as potent chitinase inhibitors. Notably, the in vitro assays show similar results to those found employing molecular docking, where compound 5 was assigned as the most potent, followed by compound 9, and then compound 7.

### Spiro-acridine derivatives interact with active site of chitinase

In order to validate the chosen approach, MD and free energy studies were applied. A set of parameters were observed during a 100 ns to analyze the stability of the three most promising chitinase-acridine systems.

**Figure 4:**
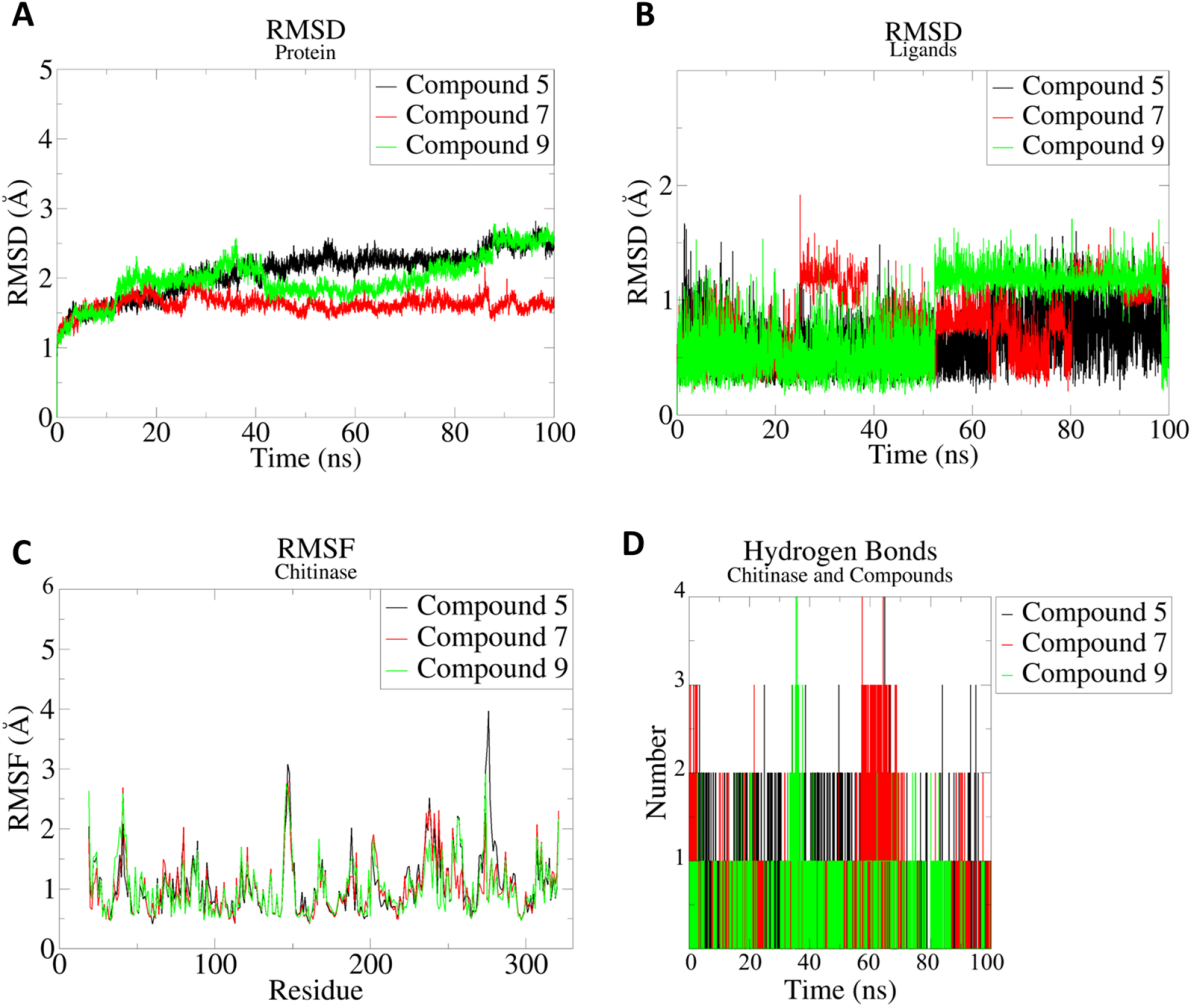
Plots of MD simulation from the chitinase-acridine systems. (**A**) RMSD of the chitinase in the three complex systems in the MD simulation at 100 ns. (**B**) RMSD of the ligands in the three complex systems in the MD simulation at 100 ns. (**C**) RMSF of the three complex systems in the MD simulation at 100 ns. (**D**) The number of H-bonds established during 100 ns MD simulations.

The evolution of RMSD from chitinase during the 100 ns is illustrated in Fig. 4A. The graph shows relative stability interspersed by fluctuations. On the other hand, in the plot of RMSD of ligands (Fig. 4B), compound 5 seems to reach a state of equilibrium, while compound 9 presents fluctuations from 50 to 100 ns. However, compound 7 presents a higher fluctuation without stabilization, from 10 to 100 ns. All compounds present a new conformation in the active site. However, compound 7 and 9 did not stabilize at the active site.

The RMSF is a parameter that provides information on the residual flexibility of the protein during its interaction with the ligand. The RMSF is illustrated in Fig. 4C, with little difference detected for the systems. The higher peak detected from amino acids 36–45, 145–150, 234–247, and 273–278 is represented by loops. The amino acids involved in the active site did not show large deviations.

Hydrogen bonds are involved with the ligand’s great connection with the receptor’s binding center and are essential for influencing ligand binding. Figure 4D displays the number of H-bonds formed during the 100 ns simulation between the compounds and chitinase. All of the investigated ligands establish hydrogen bonds with the crucial amino acids of the chitinase active site. The highest number of hydrogen interactions is observed for compound 5, with a total of four H-bonds. However, this complex is established with up to two hydrogen bonds, which emphasizes the strong attraction of these compounds to the chitinase enzyme.

The Poisson Boltzmann method estimates the binding free energy more accurately than the molecular docking approach. This method is based on three energetic types that have bonded and non-bonded interactions, polar solvation energy, and non-polar solvation energy^34^.

Results of the binding free energy calculated using the last 100 ns of the MD trajectories are represented in Table 1. These results represent the sum of the Van der Waals, electrostatic, polar salvation, and SASA energies. The binding free energy for complexes Chitinase-Compound 5, Chitinase-Compound 7, and Chitinase-Compound 9 is − 77.51 ± 10 kcal/mol, − 20.8 ± 35 kcal/mol, and − 25.32 ± 41 kcal/mol, respectively. These results clearly show that both compound 7 and 9 have significant negative energies, but compound 5 generates the best free energy, which reflects its strong binding with chitinase, and therefore reinforces the previous results of the in vitro studies.

**Table 1:** Results, showing the Van der Waals electrostatic, polar solvation, SASA, and binding energy in kcal/mol for the studied complexes.

Complex	Van der Waals	Electrostatic	Polar solvation	SASA energy	Total energy
Compound 5	− 88.50 ± 3	− 25.10 ± 5	45.35 ± 13	− 9.25 ± 0.5	− 77.51 ± 10
Compound 7	− 66.87 ± 30	− 26.14 ± 24	76.01 ± 42	− 8.32 ± 3	− 20.80 ± 35
Compound 9	− 65.52 ± 28	− 31.93 ± 26	86.07 ± 65	− 9.40 ± 3	− 25.32 ± 41

## Discussion

The IVS docking-based tool is used to screen a protein database for a query ligand. Initially conceived by Chen and Zhi^35^ in the identification of targets, the IVS method has grown over the years, with several tools available for commercial and scientific use having been reported. Studies demonstrate the efficiency of the methodology when carrying out in vitro studies with the best profile targets listed by the program^24,36^.

In docking studies, Wang et al.^37^ listed Autodock Vina as having the best scoring power. In another study, Boittier et al.^38^ compared the efficiency of various programs. Their study identified that Vina presents the best predictive values, highlighting that the program can accurately diagnose molecular features. Thus, it is observed that the IVS has been shown to be a powerful and robust tool in drug development.

The use of IVS in our research identified fungal chitinases as the most potent biological target for the selected dataset of acridine derivatives. In addition to identifying in silico inhibitory activity against fungal chitinase (*A. fumigatus*), this study indicated putative activity on bacterial chitinases from *S. marcescens* (Table S1). So far, no inhibitory studies of acridine derivatives with chitinases have been reported. Thus, this is the first evidentiary report.

The characteristic GH18 DXDXE domain was identified in all tested chitinases and monitored during simulations, as it is essential for chitinolytic activity. This motif was also found to have a good druggable score. Additionally, strong chitinase-acridine interactions can affect chitinase interaction with its native substrate, which suggests these compounds act as protein inhibitors. It was observed through simulations of the stability of interaction of the acridine compounds and chitinase structures tested that compounds 5, 7 and 9 are fungal chitinase inhibitors.

Argifin, reported as a cyclic peptide chitinase inhibitor, was an efficient inhibitor of *Lucilia cuprina* and chitinase B from *S. marcescens*^39,40^. From the pentapeptide, a tripeptide (VR0) portion was identified as the active fragment of the GH18 family inhibitor^41^. Recently, Souza et al.^12^ identified the activity of the plumieridine compound using the methodology of computational ligand screening to investigate the possible biological target. Using in vitro biological evidence, plumieridine showed inhibitory activity for *Cryptococcus neoformans* growth, which was attributed to the inhibition of chitinolytic activity.

From this perspective, the conformation and interactions of our acridine derivatives was similar to the inhibitors in the active site, which may indicate a possible mimicry of activity. Computational studies performed here showed that compound 5 was the most potent inhibitor, followed by compound 7 and 9. IVS pointed to *Af*ChiB1 as a potential target, as chitinases from *T. harzianum* presented high sequence identity and structural similarity. As they are commercially available, the model organisms of chitinases were used in in vitro assays. Inhibition of *T. harzianum* chitinolytic activity by the tested acridine compounds corroborates with in silico findings, where the IC_50_ was 0.6 ng/µL for compound 5, 5.7 ng/µL for compound 7 and 1.8 ng/µL for compound 9. Although compound 5 was observed to have the best activity, solubility in aqueous solution is a known issue for acridine compounds.

Overall, through using IVS, fungal and bacterial chitinases were identified as potential targets for acridine derivatives. To validate this result, a series of in silico experiments were conducted to shed light on the atomic level interaction between inhibitor and target protein. Finally, the dual-target activity of acridine derivatives was observed first for DNA topoisomerase II for antitumor activity^28^ and now chitinases, using *T. harzianum* as a model. Future perspectives lie in using compound 5 as a scaffold for the development of stronger and specific chitinase inhibitors that can, potentially, be used in in vivo experiments with model organisms.

## Methodology

### Dataset

**Figure 5:**
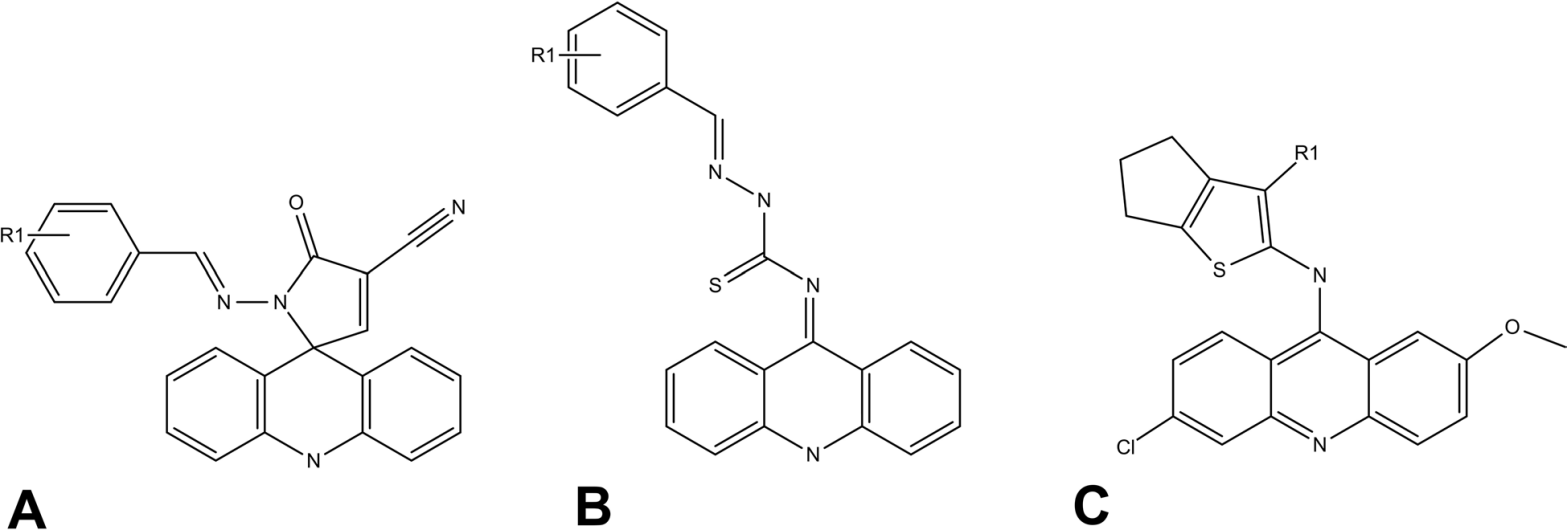
Acridine core of datasets. (**A**) spiro-acridine (**B**) thiosemicarbazone-acridine and (**C**) thiophene-acridine.

In our research group, a total of 28 acridine derivatives were selected from a recent publication, with 3 classes of acridine derivatives (Fig. 5): spiro-acridine^27,29^, thiosemicarbazone-acridine^28^, and thiophene-acridine^18^. All names, structures and references are presented in Supplementary Table S4. The structural characterizations and cytotoxicity studies were published by the authors.

The two-dimensional structures of compounds in this series were created using the MarvinSketch 19.21 program, 2019, ChemAxon (http://www.chemaxon.com), considering a pH of 7.4 for protonation. All possible stereoisomers were created for each molecular model. All models were cured and edited under inspection of the Avogadro program^42^. MOPAC software was used to optimize each molecular geometry employing the PM7 semi-empirical method^43^. The COSMO approach enabled the simulation of bulky solvation behavior^44^.

### Inverse virtual screening

A representative compound of the series (compound 1, Table S4) was selected to perform an inverse virtual screening (target fishing) to help expose possible biological activity for the series of compounds. Compound 1 was selected for the IVS analysis due the best biological activity value of the series. In order to carry out target fishing, a non-redundant library of targets, comprising about 23,000 structures, was created. Such targets are derived from the Protein Data Bank^45^ and originally contained ligands bound to them. All bound ligands were removed, and proper inputs were created to perform automated docking simulation with Autodock Vina^46^. Ad hoc scripts were created and used for automation (Fig. S5). All script data are included in the compressed file (PDBQT_TARGETS_ALL.zip.), available in the supplementary material of this research. The data was sorted according to the scores and analyzed by human inspection to determine the priority order of possible biological tests.

### Protein alignment

The chitinase B from *A. fumigatus*, obtained from IVS screening, was used to align with *T. harzianum* sequences. The chitinase structure from *T. harzianum* was used as a model organism because it is commercially available and has similar structures to the chitinase from the GH18 family^12^. In this way, chitinase from *T. harzianum* was obtained from the Protein Data Bank under the access codes 6EPB (Chit42) and 7ZYA (Chit33). Thus, chitinase protein sequences were downloaded from RCSB PDB (Table 2). Multiple sequence alignment was created via ClustalO in Jalview^47^.

**Table 2:** Identification of chitinase proteins used in sequence alignment.

Protein	PDB ID	Ligand	Organism	Reference
Chitinase B	2A3B	Caffeine	*A. fumigatus*	^14^
Chit33	7ZYA	2-amino-2-hydroxymethyl-propane-1,3-diol	*T. harzianum*	^48^
Chit42	6EPB	1,2-ethanediol	*T. harzianum*	^49^

### Binding site optimization for molecular docking

The protein structures were subjected to preparation by deleting solvents, adding hydrogens, charges, and replacing the rotamer library with incomplete side chains in the Chimera UCSF program^50^. Chitinases from *A. fumigatus* and *T. harzianum* had ligands docked to the binding sites with AutoDock Vina 1.1.2 to predict binding poses and scores^46^. The 28 acridine derivatives, present in our research group (Table S4), were used to assess their potential as an inhibitor of the chitinases studied. The redocking results obtained from Vina were used as input data in AutoDockTools 1.5.7^51^, and the crystallized PDB binder was used as a fixed reference. The RMSD was calculated, using the AtomName matcher as a parameter. Molecular docking was performed according to Rao et al.^14^, where the inhibitor caffeine (CFF) position in the reference PDB ID 2A3B is considered as the initial position. This active site position is shown by the domains of the Glycoside Hydrolase 18 superfamily. Protein preparation (addition of hydrogens) and ligand placement in the active site were achieved using UCSF Chimera interface^50^. The plumieridine inhibitor was also used as a comparative compound due to its activity against *T. harzianum*^12^.

### Chitinolytic activity assays

In vitro tests were performed to validate the computational methods. The chitinolytic activity was performed using 4-methylumbelliferyl-β-d-N,N′,N″-triacetylchitotrioside (Sigma-Aldrich Co.) as substrate. A standard curve was constructed using 4-methylumbelliferyl (4MU) (Sigma-Aldrich Co.). Assays were performed in 96-well coated microplates (Greiner CELLSTAR^®^ Sigma-Aldrich Co.) and consisted of 95 µL McIlvaine buffer pH 6.0, 5 µL of the substrate (0.8 mM), 5 µL Lysing Enzymes from *T. harzianum* (200 mg/mL in PBS 1 x; Sigma-Aldrich Co.), and 5 µL of the evaluated compound. The reaction was incubated at 37 °C for 15 min. Fluorescence was measured at 355 nm excitation and 460 nm emission using SpectraMax M3. The inhibitory assays were performed with increasing concentrations of the compounds evaluated (compounds 5, 7, and 9) diluted in DMSO. The different concentrations of each compound were used according to the indicated solubility^28^. The final concentrations of compound 5 in the assay were: 0.568 ng/µL, 1.13 ng/µL, and 2.27 ng/µL; compound 7 final concentrations were: 5.68 ng/µL, 11.3 ng/µL, and 22.7 ng/µL; and compound 9 final concentrations were: 2.84 ng/µL, 5.68 ng/µL, and 11.3 ng/µL. Quantification of the samples was based on the relative fluorescence units (RFU) using the previously established standard curve^12,52^.

### Molecular dynamic simulations

All simulations were carried out using the GROMACS Simulation package version 5^53^ and CHARMM force field^54^ for chitinase of *T. harzianum*. The compounds 5, 7 and 9 had their topology built using SwissParam^55^.

The solvent properties were emulated using the TIP3P water model with a cubic box large enough to allow a minimum of 1.0 nm space from the protein to the box walls^56^. The system charge was neutralized with the addition of ions at the physiological concentration of 0.15 mM. Geometry optimization of the solvated system was performed using the steepest descent algorithm (5000 steps), followed by equilibration simulations with nVT and nPT ensembles keeping the inhibitor and the protein restrained. The temperature was kept at 300 K coupling the system to a V-rescale thermostat (0.1 ps), while the pressure was also kept constant at 1 bar using the Parinello-Rahman coupling algorithm.

The short range Coulombic and Lennard–Jones interaction energies between compounds and the surroundings were monitored during the productive simulation step. The molecular dynamics simulation was performed for a run time of 100 ns. Coulomb and van der Waals interactions within a shorter-range cutoff of 1.0 nm were computed every time step. Particle Mesh Ewald was employed to minimize the effects of truncating the electrostatic interactions beyond the 1.2 nm long-range cutoff^57^. Covalent bonds in the protein were constrained using the LINCS algorithm^58^.

To evaluate protein–ligand interactions we used RMSD (Root Mean Square Deviation), RMSF (Root Mean Square Fluctuation), and number of Hydrogen bonds (H-bond). The VMD 1.9.2 software^59^ was used to visualize the MD simulation, and RMSD and RMSF plots were generated with xmgrace^60^.

Binding free energy calculations use the g_mmpbsa^61^ tool. MMPBSA is widely implemented in drug discovery to estimate the binding affinities of small molecule interactions with their biomolecular targets. All scripts for MMPBSA calculations were obtained from http://rashmikumari.github.io/g_mmpbsa/.

## Conclusion

In this study the results presented in the inverse virtual screening for acridine derivatives helped us to find that chitinase enzyme is the best target for these derivatives. With the help of computational tools, such as molecular docking data, it was possible to identify the compounds with probable inhibitory activity for chitinase. The three classes of acridine derivatives, the spiro-acridines, were the most promising. Moreover, three derivative compounds showed activity prediction and high docking score, being confirmed in vitro, with compound 5 showing the best profile with an IC_50_ of 0.6 ng/µL. Additionally, molecular dynamic simulation and free energy validated the chosen approach by identifying the stability in the formation of the chitinase-compound 5 complex. Thus, with this analysis, we increase the probability of selecting more potentially active molecules using structure based virtual screening approaches.

## Supplementary Information

Supplementary Information 1.

Supplementary Information 2.

## Data Availability

All data generated or analyzed during this study are included in this published article (and its Supplementary Information files). The datasets and codes generated during the current study are available from the corresponding author on reasonable request.
