# Supplementary material for: Scaffold repositioning of spiro-acridine derivatives as fungi chitinase inhibitor by target fishing and in vitro studies
Source: Sci Rep. 2023 May 5;13:7320. doi: 10.1038/s41598-023-33279-9 (PMC10163251; doi:10.1038/s41598-023-33279-9)
Supplement: Supplementary file 1 — Supplementary Information 1. [file 41598_2023_33279_MOESM1_ESM.docx]

Supporting information

**Scaffold repositioning of spiro-acridine derivatives as fungi chitinase inhibitor by target fishing and *in vitro* studies**

Jéssika de Oliveira Viana^a^, Éden Silva e Souza^b^, Nicolau Sbaraini^c^, Marilene Henning Vainstein^c^, Joilly Nilce Santana Gomes^d^, Ricardo Olímpio de Moura^d^, Euzébio Guimarães Barbosa^a,e^*

*^a^ Post-graduate Program in Bioinformatics, Bioinformatics Multidisciplinary Environment, Federal University of Rio Grande do Norte, Natal, Brazil.*

*^b^ School of Biomolecular and Biomedical Science & BiOrbic - Bioeconomy Research Center, University College Dublin, Ireland.*

*^c^ Biotechnology Center, Postgraduate Program in Cellular and Molecular Biology, Federal University of Rio Grande do Sul, Porto Alegre, Brazil.*

*^d^ Department of Biological Sciences, State University of Paraíba, Campina Grande, Brazil.*

*^e^ Post-graduate Program in Pharmaceutical Sciences, Faculty of Pharmacy, Federal University of Rio Grande do Norte, Natal, Brazil.*

** Corresponding author: Email address* [*euzebiogb@gmail.com*](mailto:euzebiogb@gmail.com)

| **Table of contents** | | |
| --- | --- | --- |
| 1 | Tables S1-S4 mentioned as supplementary material. | 2 - 11 |
| 2 | Figures S1 - S5 mentioned as supplementary material. | 12 - 16 |

**Table S1.**  The best targets obtained in IVS prediction. The score is present in kcal/mol.

| **Protein** | **PDB ID** | **Ligand** | **Organism** | **Score** |
| --- | --- | --- | --- | --- |
| Chitinase B | 2A3B | Caffeine | *Aspergillus fumigatus* | -10.6 |
| Chitinase B | 4Z2I | Macrolide inhibitor | *Serratia marcescens* | -10.4 |
| Chitinase B | 3WD1 | syn-triazole inhibitor | *Serratia marcescens* | -10.3 |
| Pteridine Reductase 1 | 2QHX | FE1 inhibitor | *Leishmania major* | -10.1 |
| Chitinase B | 4Z2G | Macrolide inhibitor | *Serratia marcescens* | -9.6 |
| Chitinase B | 4Z2J | Macrolide inhibitor | *Serratia marcescens* | -9.5 |
| Chitinase B | 4Z2L | Macrolide inhibitor | *Serratia marcescens* | -9.4 |
| Chitinase | 3WQV | Piperazine | *Ostrinia furnacalis* | -9.4 |
| Chitinase A | 3ARP | Dequalinium | *Vibrio harveyi* | -9.3 |
| Chitinase B | 3WD2 | Azide inhibitor | *Serratia marcescens* | -9.2 |
| Chitinase B | 4Z2K | Macrolide inhibitor | *Serratia marcescens* | -9.2 |

**Table S2.** Docking score of acridine derivatives for *A. fumigatus*. All scores are presented in kcal/mol.

| **Compounds** | ***Af*ChiB** |
| --- | --- |
| 1 | -8.5 |
| 2 | -9.3 |
| 3 | -9.4 |
| 4 | -9.4 |
| 5 | -10.9 |
| 6 | -9.1 |
| 7 | -10.1 |
| 8 | -9.3 |
| 9 | -10.6 |
| 10 | -8.9 |
| 11 | -10.1 |
| 12 | -10.2 |
| 13 | -9.2 |
| 14 | -10.1 |
| 15 | -9.9 |
| 16 | -10.0 |
| 17 | -10.2 |
| 18 | -10.0 |
| 19 | -10.2 |
| 20 | -9.6 |
| 21 | -9.0 |
| 22 | -9.1 |
| 23 | -9.0 |
| 24 | -9.9 |
| 25 | -9.3 |
| 26 | -10.0 |
| 27 | -8.9 |
| 28 | -9.4 |
| Caffeine | -6.3 |

**Table S3.** Docking score of acridine derivatives for *T. harzianum.* All scores are presented in kcal/mol.

| **Compounds** | **Chit42** | **Chit33** |
| --- | --- | --- |
| 1 | -9.1 | -7.5 |
| 2 | -8.7 | -5.9 |
| 3 | -9.2 | -7.6 |
| 4 | -9.5 | -7.4 |
| 5 | -9.8 | -7.8 |
| 6 | -9.5 | -7.4 |
| 7 | -9.5 | -7.3 |
| 8 | -9.3 | -7.4 |
| 9 | -9.3 | -7.7 |
| 10 | -9.2 | -7.3 |
| 11 | -9 | -6.8 |
| 12 | -9.3 | -6.8 |
| 13 | -9.1 | -6.1 |
| 14 | -8.8 | -7.1 |
| 15 | -8.3 | -7.2 |
| 16 | -8.1 | -7.2 |
| 17 | -8.3 | -6.9 |
| 18 | -8 | -6.7 |
| 19 | -8 | -6.8 |
| 20 | -9.2 | -6.8 |
| 21 | -9.5 | -6.8 |
| 22 | -8.7 | -6 |
| 23 | -0.5 | -7.1 |
| 24 | -9.3 | -7.2 |
| 25 | -9.2 | -6.7 |
| 26 | -9.3 | -7.2 |
| 27 | -9.5 | -6.4 |
| 28 | -8.7 | -7.1 |
| Plumieridine | -7.5 | -6.6 |

**Table S4.** Acridine derivatives listed in this study.

| **Name** | **Structure** | **IUPAC Name** | **Reference** |
| --- | --- | --- | --- |
| 1 | 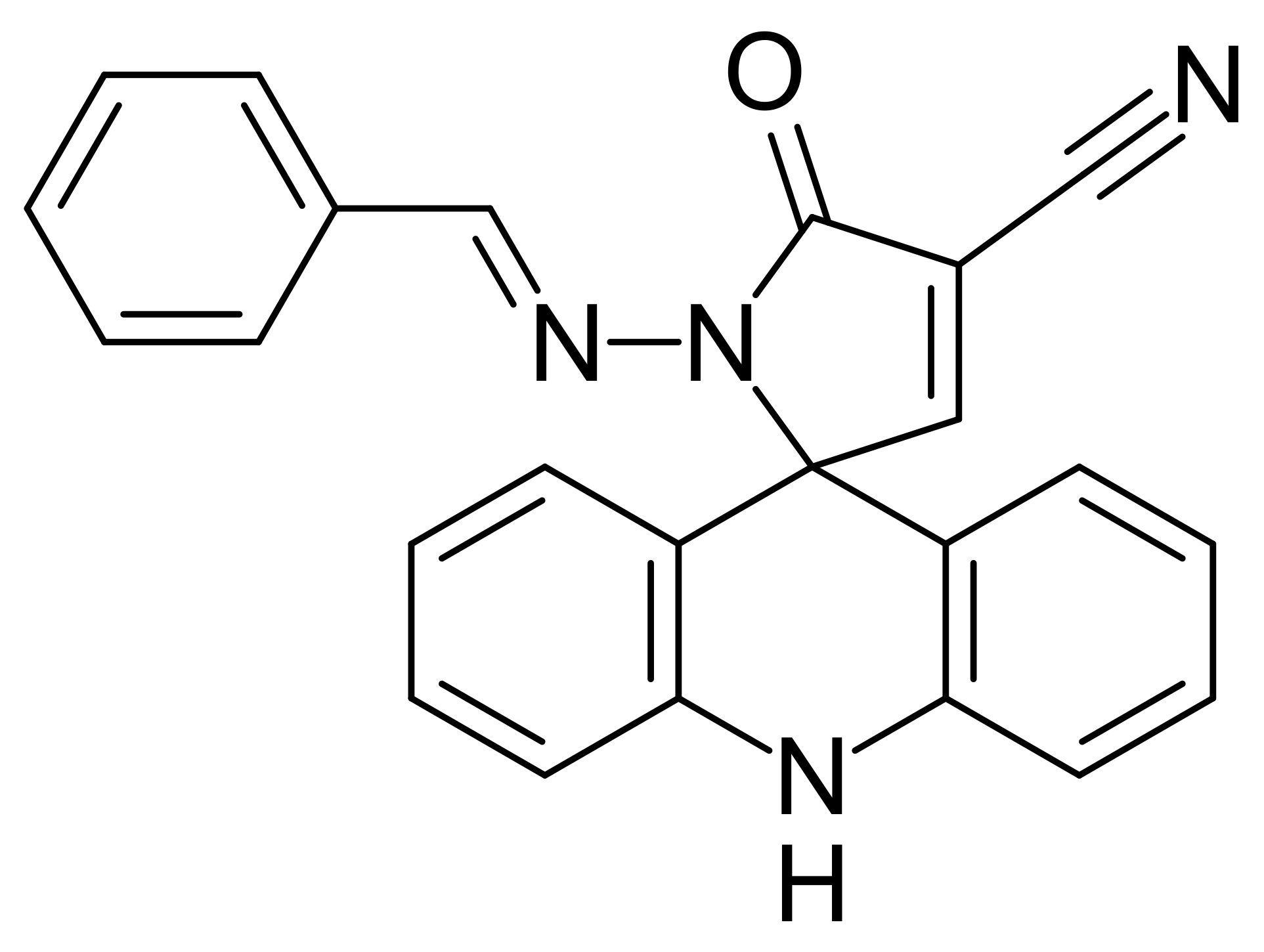 | (E)-1 -(benzylideneamino)-5 -oxo-1 ,5 -dihydro-10Hspiro[acridine-9,2 -pyrrole]-4 -carbonitrile | [26] |
| 2 | 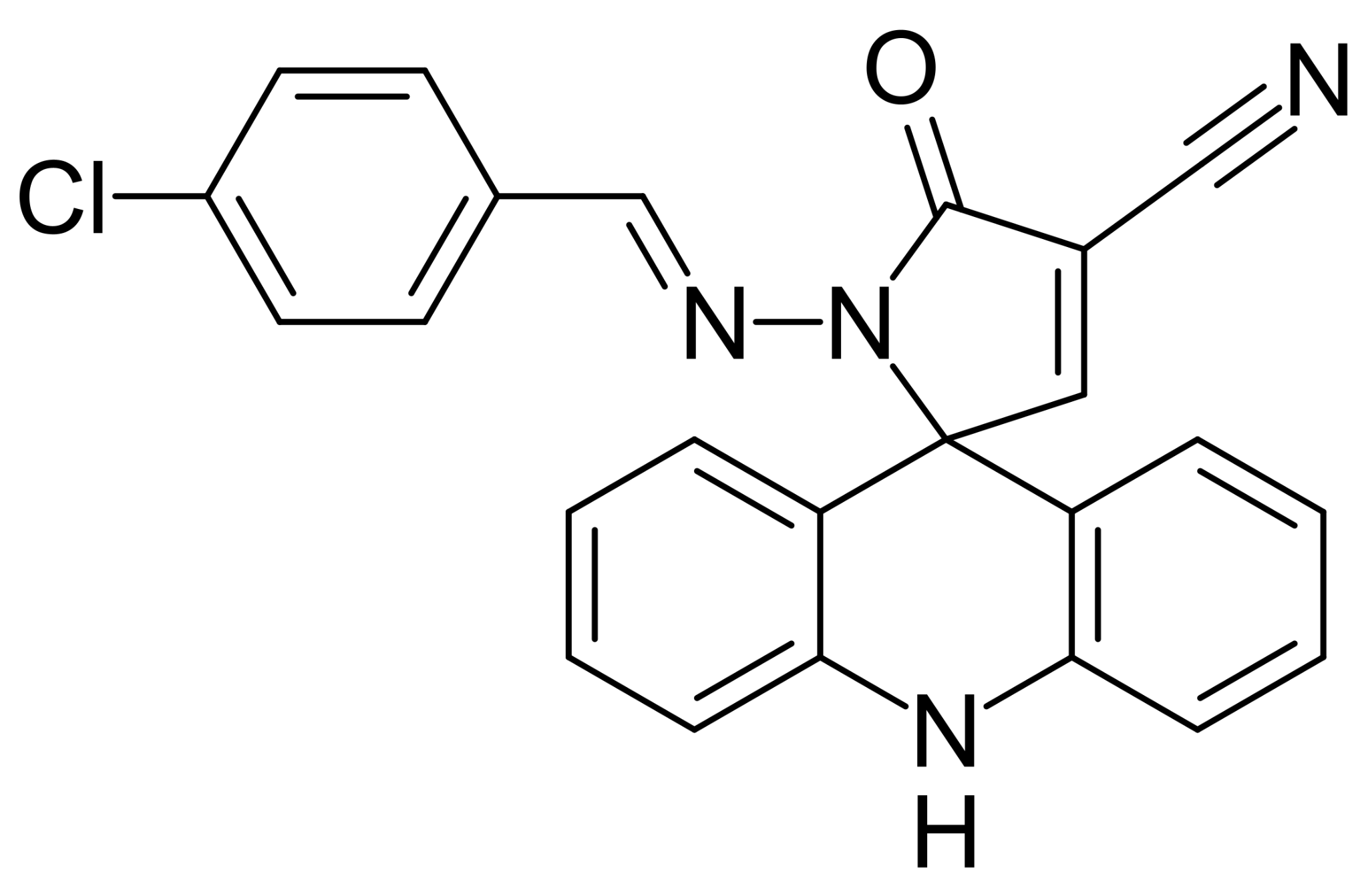 | 1-(4-chlorobenzylideneamino)-5-oxo-1,5-dihydro-10H-spiro[acridine-9,2- pyrrole]-4-carbonitrile | [26] |
| 3 | 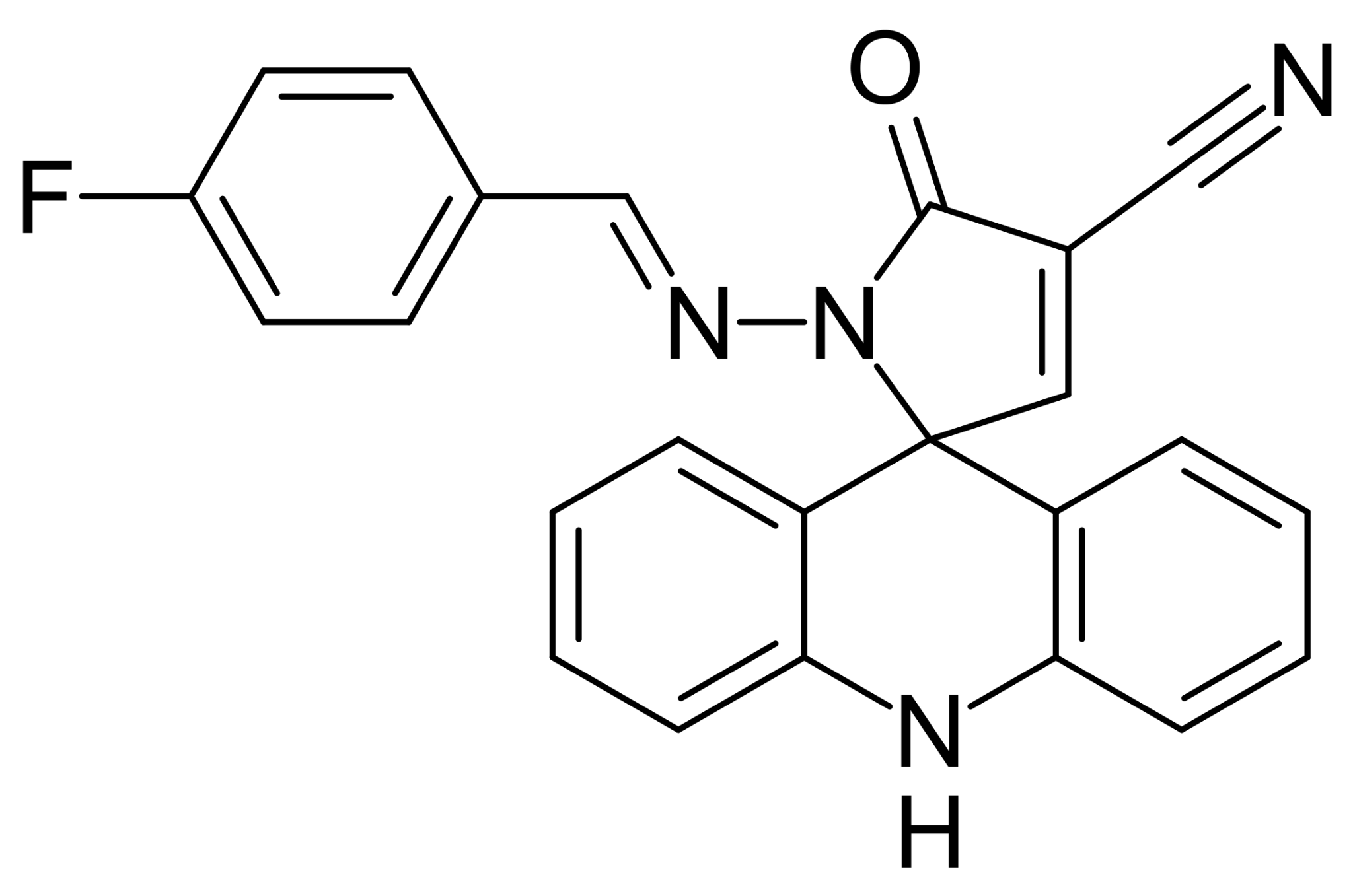 | 1-(4-fluorobenzylideneamino)-5'-oxo-1,5-dihydro-10H-spiro[acridine-9,2- pyrrole]-4-carbonitrile | [26] |
| 4 | 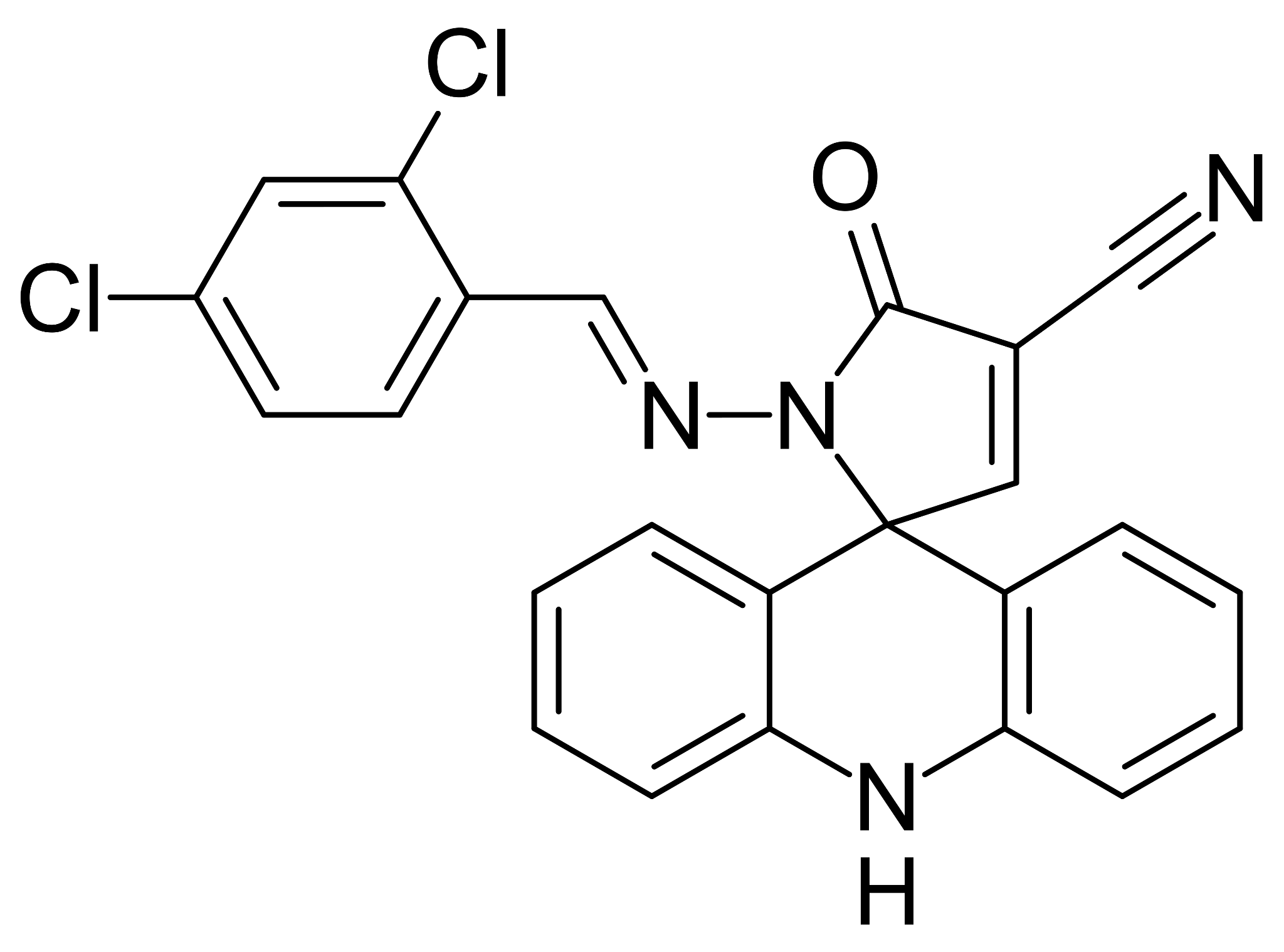 | 1-(2,4-dichlorobenzylideneamino)-5-oxo-1,5-dihydro-10H-spiro[acridine-9,2- pyrrole]-4-carbonitrile | [26] |
| 5 | 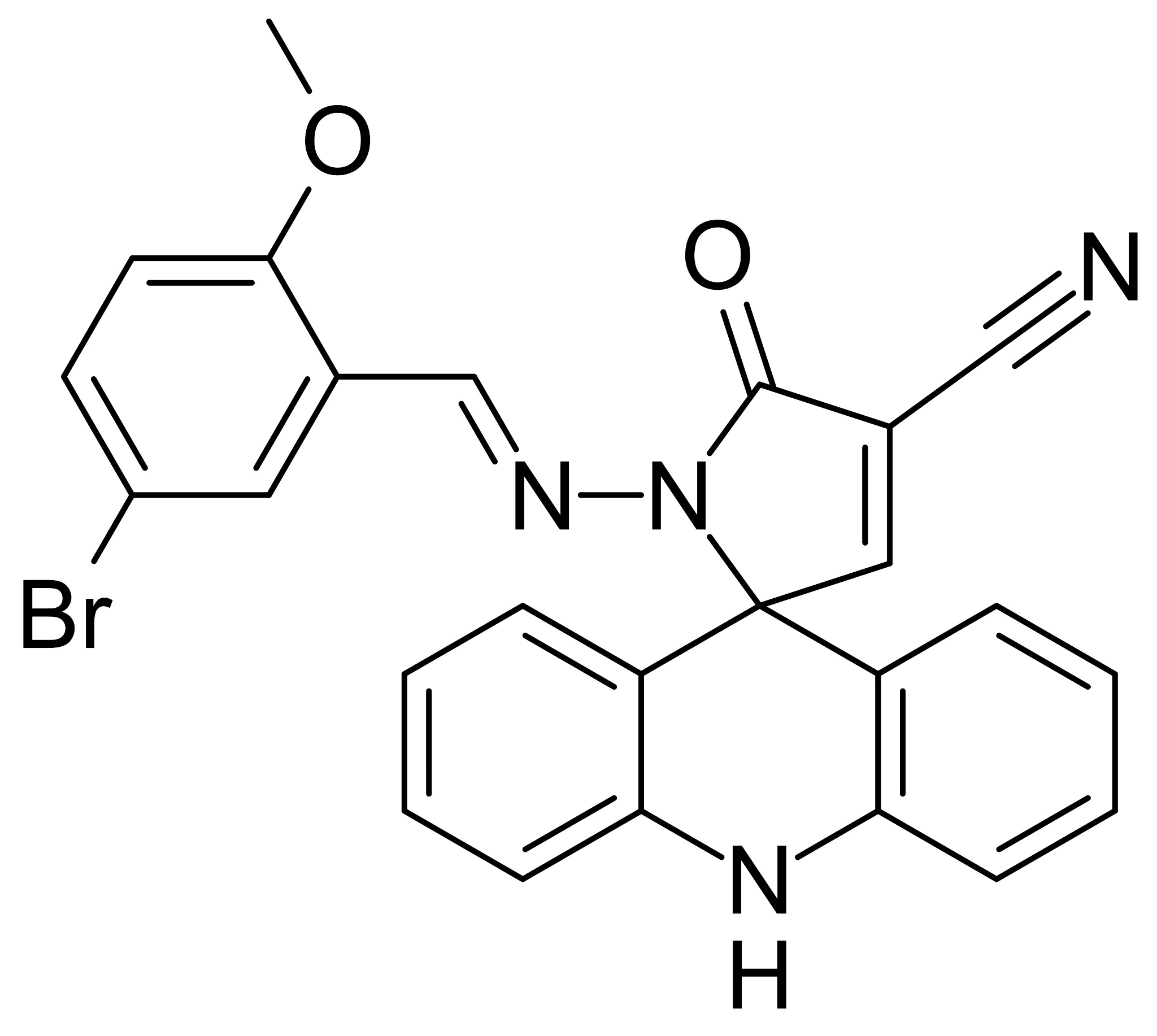 | 1-(5-bromo-2-methoxybenzylideneamino)-5-oxo-1,5-dihydro-10H-spiro[acridine9,2-pyrrole]-4-carbonitrile | [26] |
| 6 | 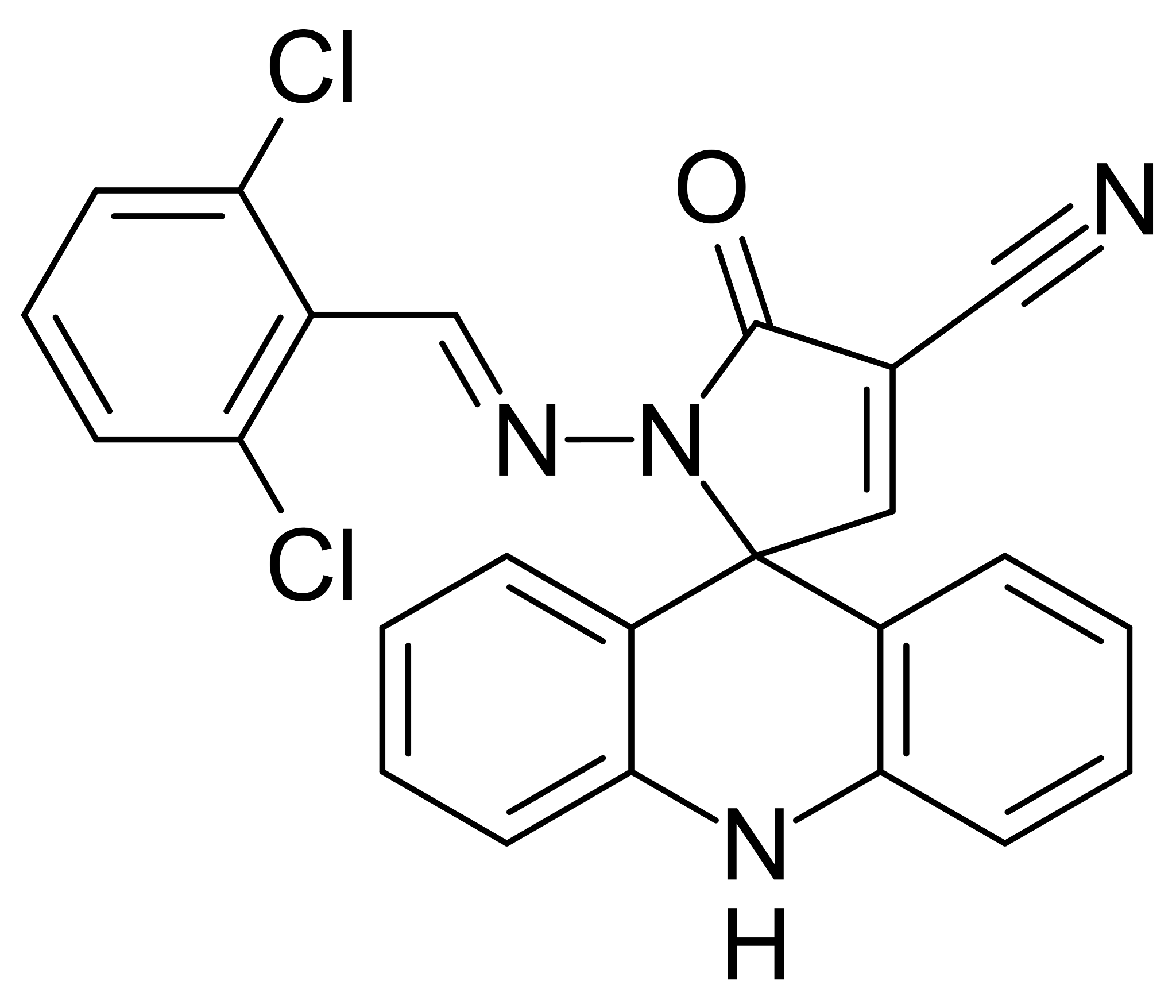 | 1-(2,6-dichlorobenzylideneamino)-5-oxo-1,5-dihydro-10H-spiro[acridine-9,2- pyrrole]-4-carbonitrile | [26] |
| 7 | 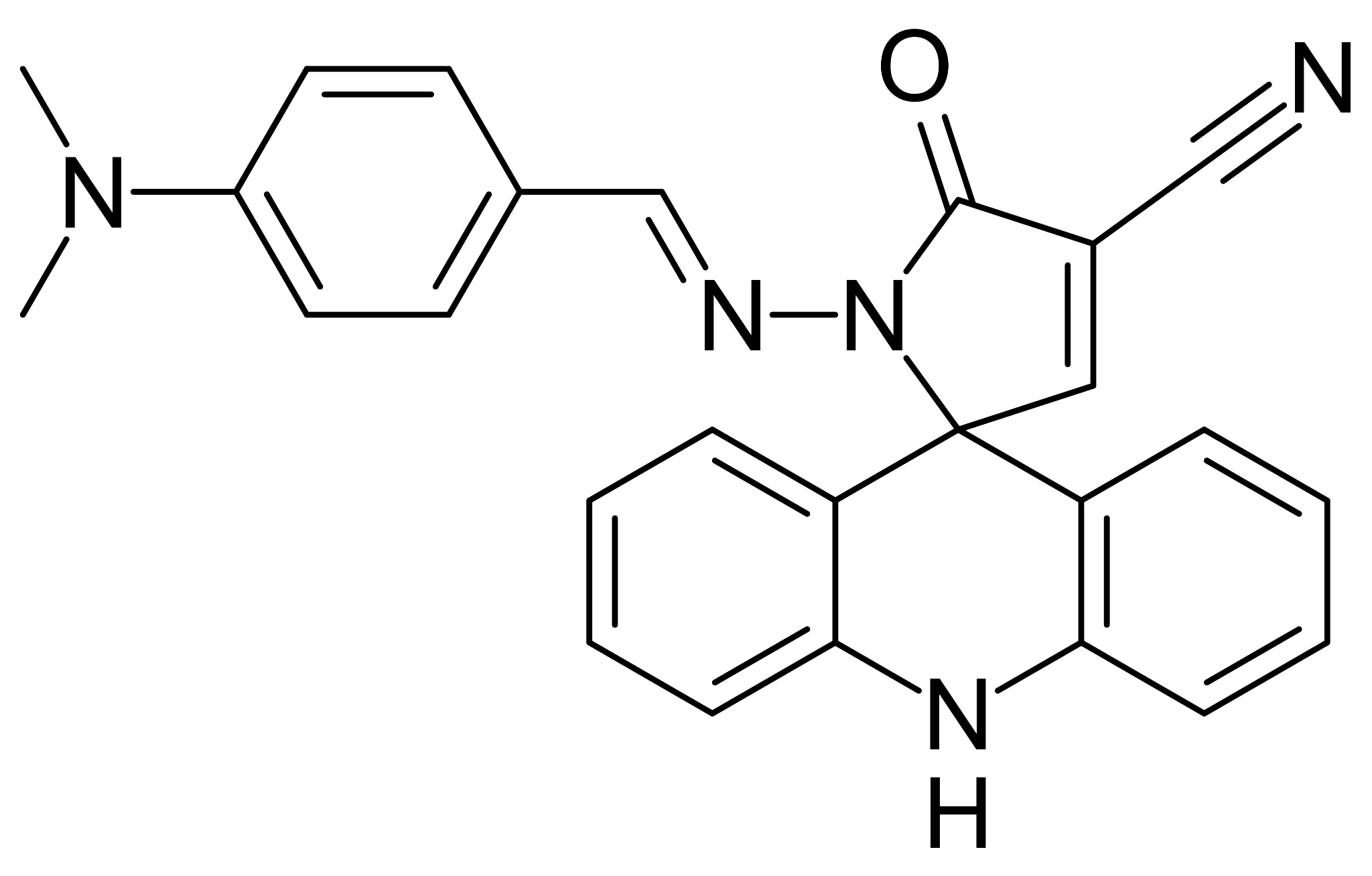 | 1-(4-dimethylaminobenzylideneamino)-5-oxo-1,5-dihydro-10H-spiro[acridine9,2-pyrrole]-4-carbonitrile | [26] |
| 8 | 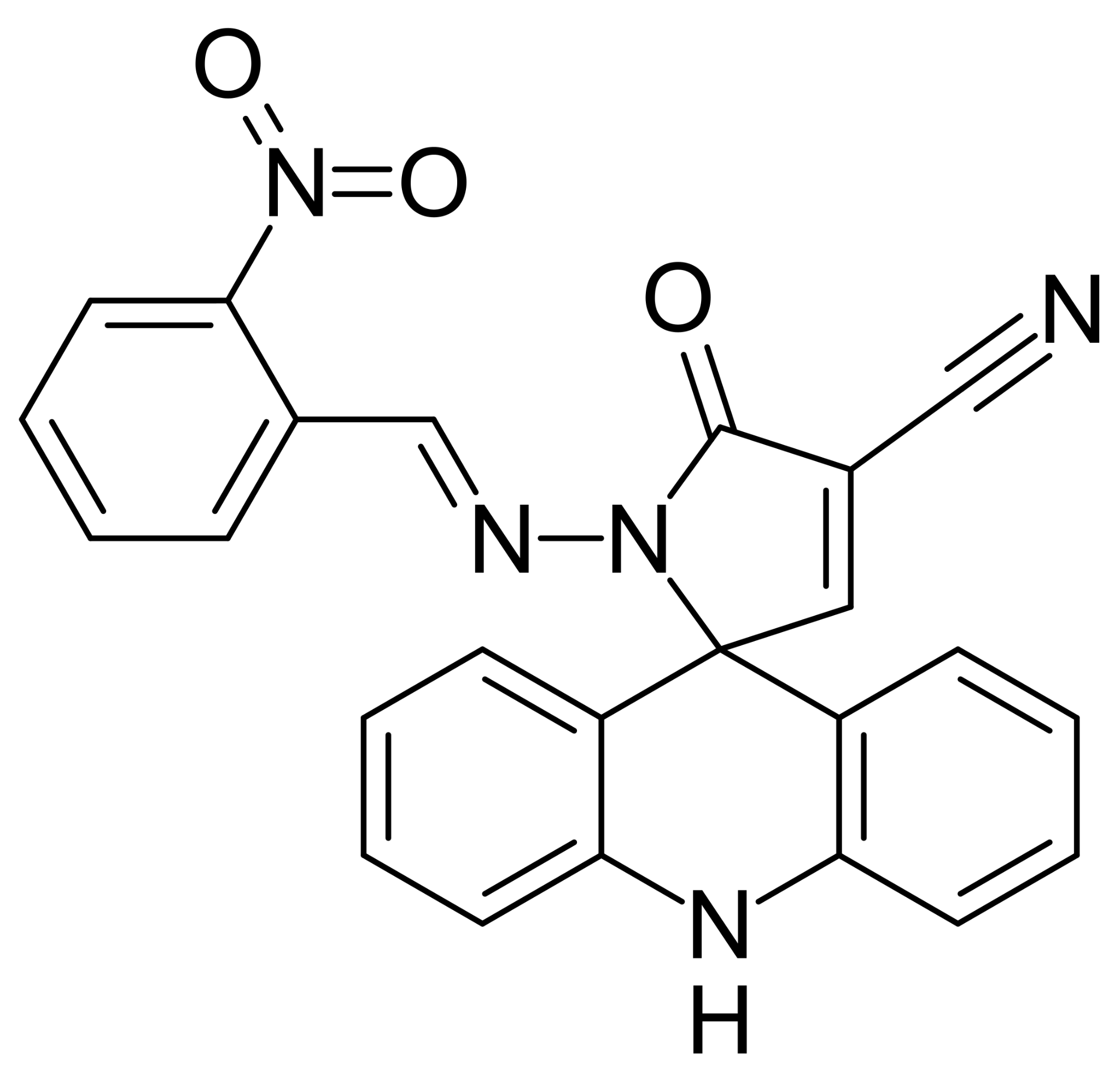 | 1-(2-nitrobenzylideneamino)-5-oxo-1,5-dihydro-10H-spiro[acridine-9,2-pyrrole]- 4-carbonitril | [26] |
| 9 | 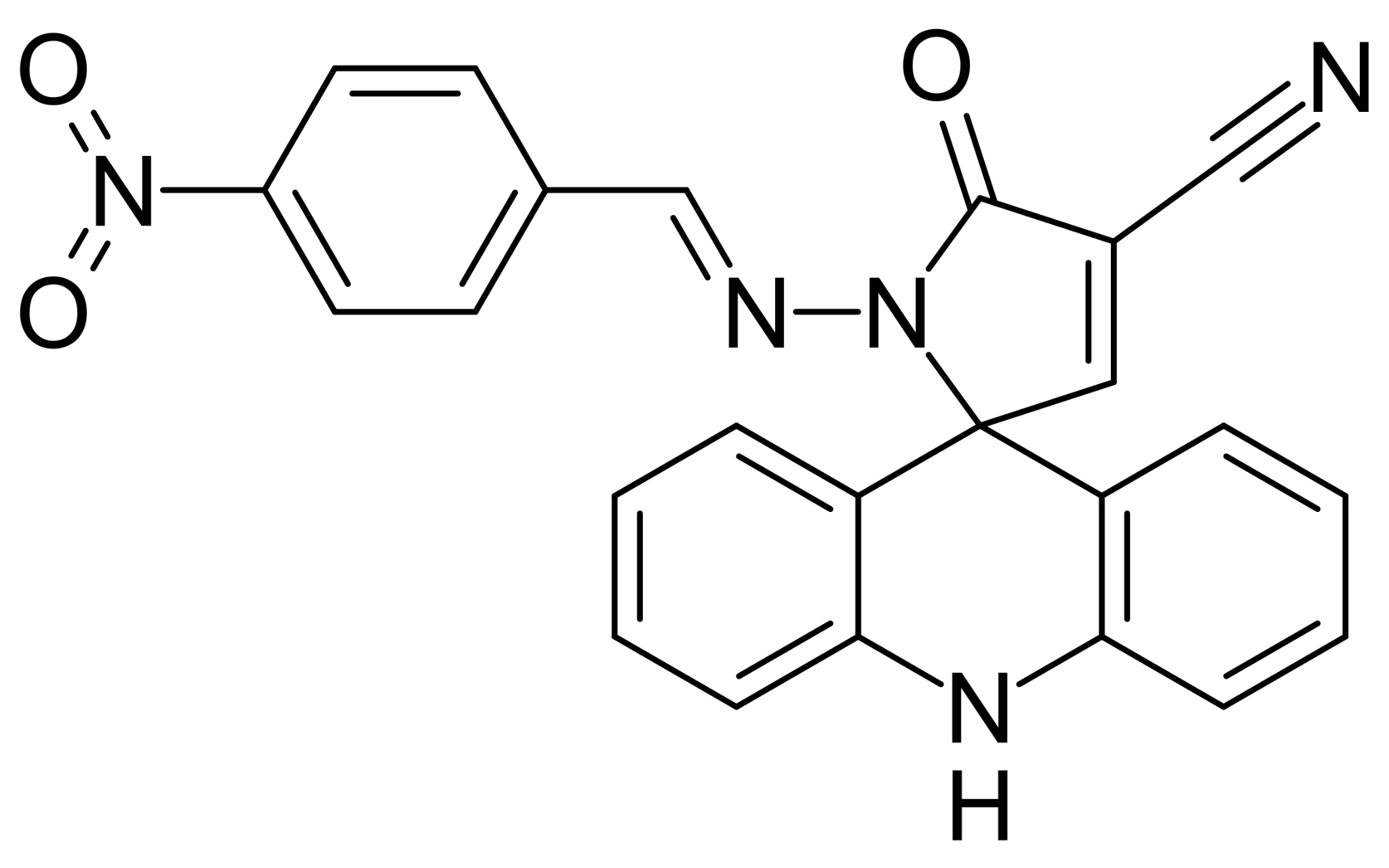 | 1-(4-nitrobenzylideneamino)-5-oxo-1,5-dihydro-10H-spiro[acridine-9,2-pyrrole]- 4-carbonitril | [26] |
| 10 | 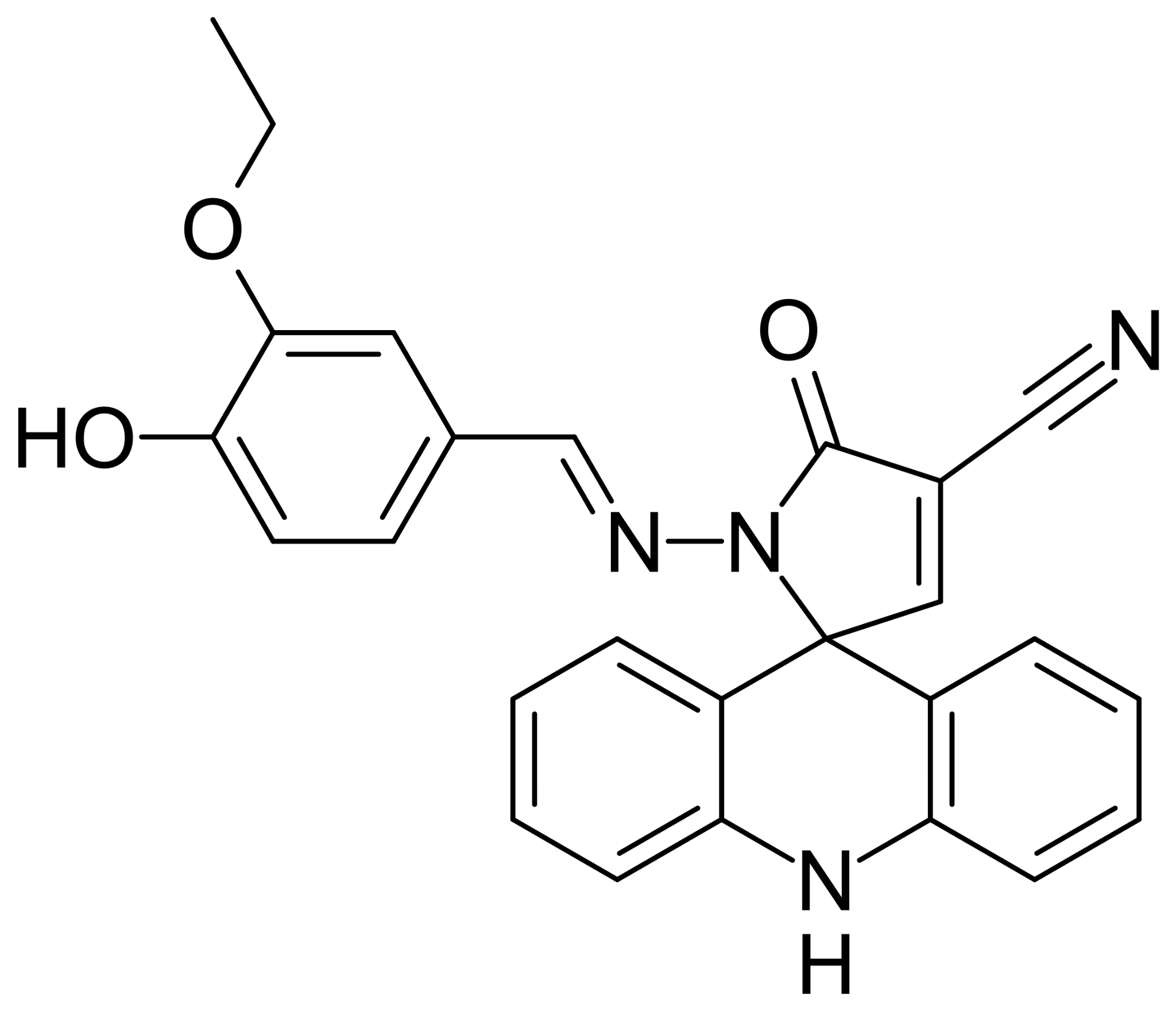 | 1-(3-ethoxy-4-hydroxybenzylideneamino)-5-oxo-1,5-dihydro-10H-spiro[acridine9,2-pyrrole]-4-carbonitrile | [26] |
| 11 | 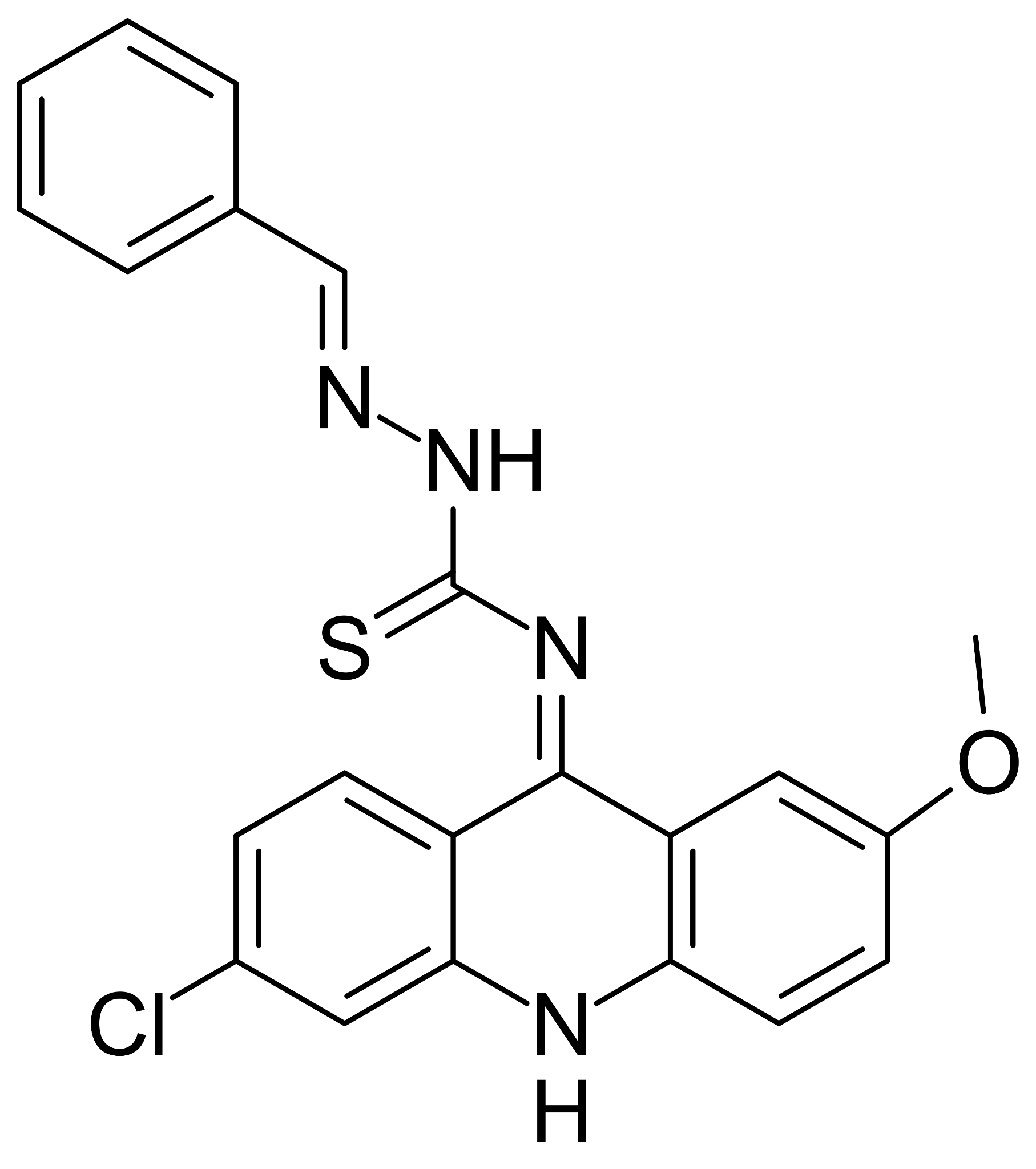 | (2E,NE)-2-Benzylidene-N-(6-chloro-2-methoxycridin-9(10H)-ylidene)hydrazinecarbothioamide | [27] |
| 12 | 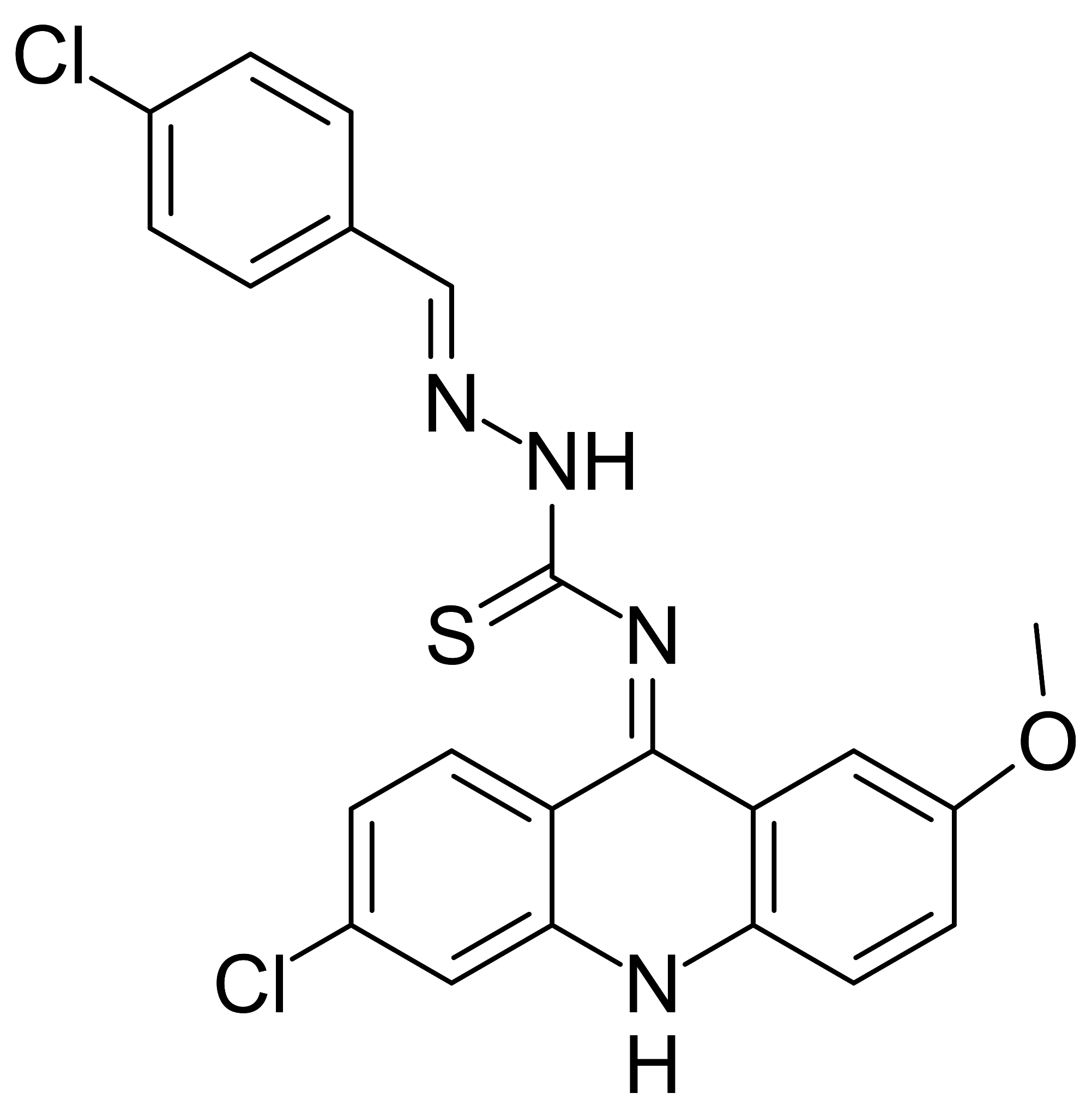 | (2E,NE)-N-(6-chloro-2-methoxycridin-9(10H)-ylidene)-2-(4-chlorobenzylidene)hydrazinecarbothioamide | [27] |
| 13 | 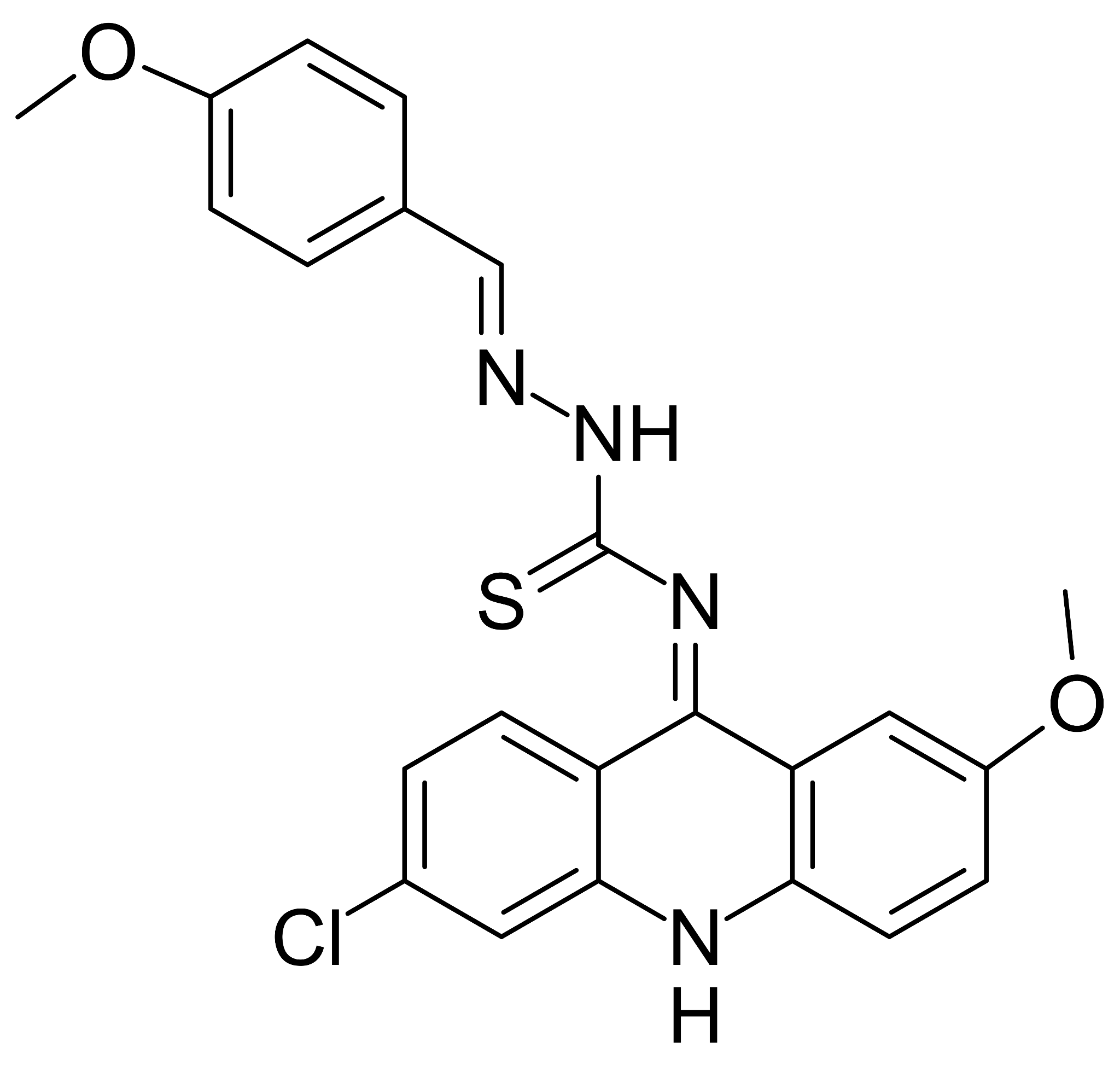 | (2E,NE)-N-(6-chloro-2-methoxycridin-9(10H)-ylidene)-2-(4-methoxybenzylidene)hydrazinecarbothioamide | [27] |
| 14 | 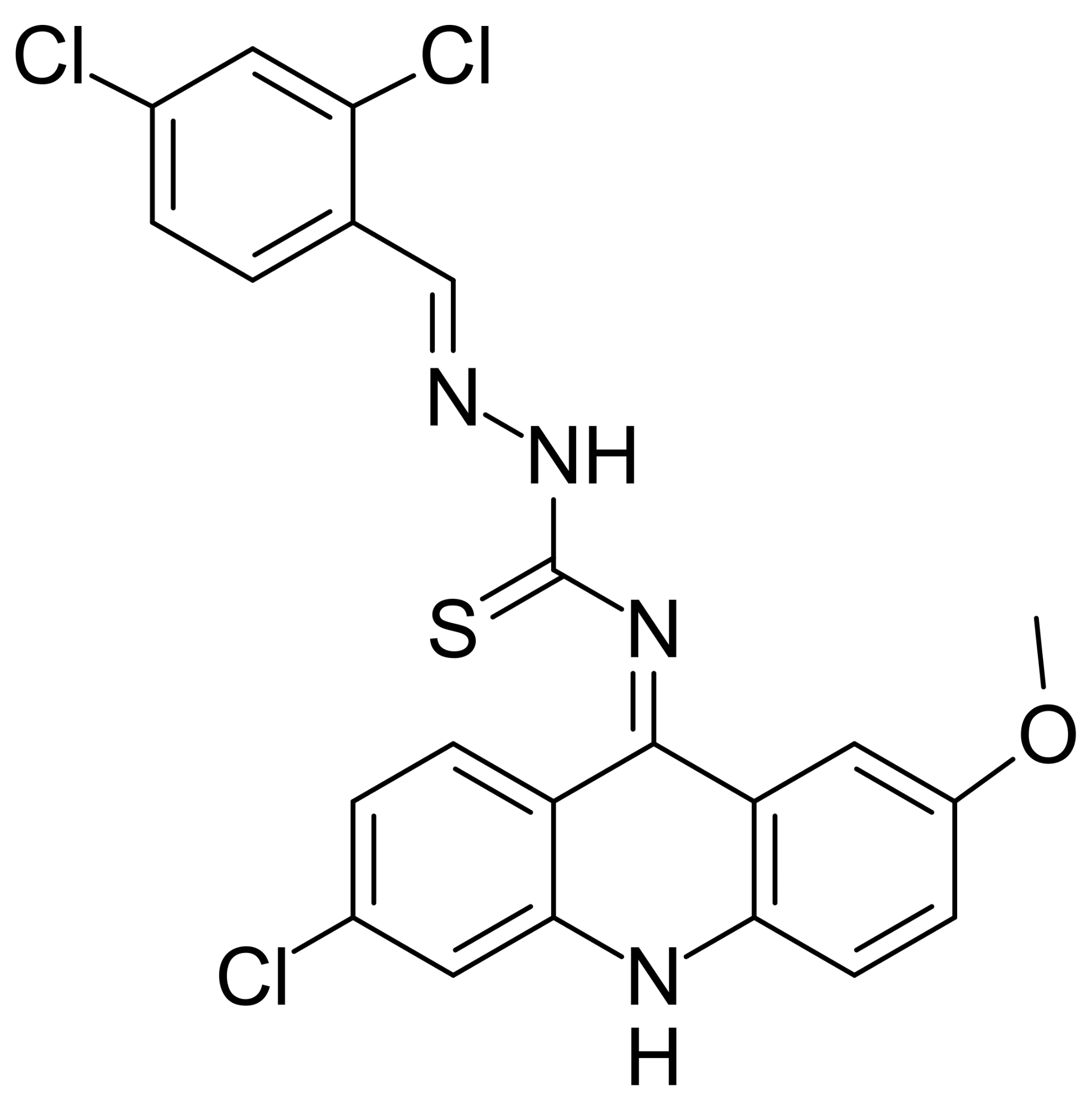 | (2E,NE)-N-(6-chloro-2-methoxycridin-9(10H)-ylidene)-2-(4-dichlorobenzylidene)hydrazinecarbothioamide | [27] |
| 15 | 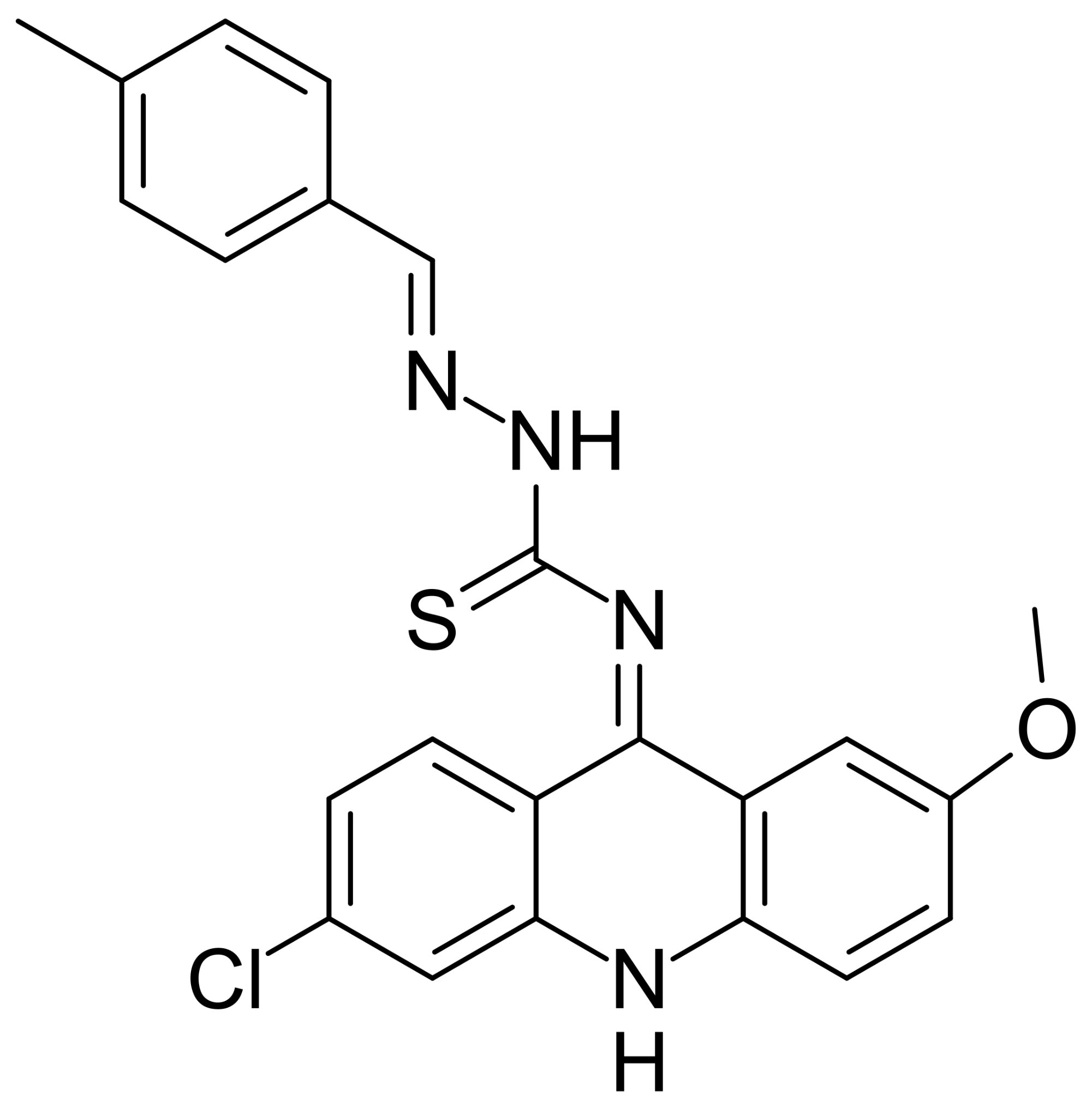 | (2E,NE)-N-(6-chloro-2-methoxycridin-9(10H)-ylidene)-2-(4-methylbenzylidene)hydrazinecarbothioamide | [27] |
| 16 | 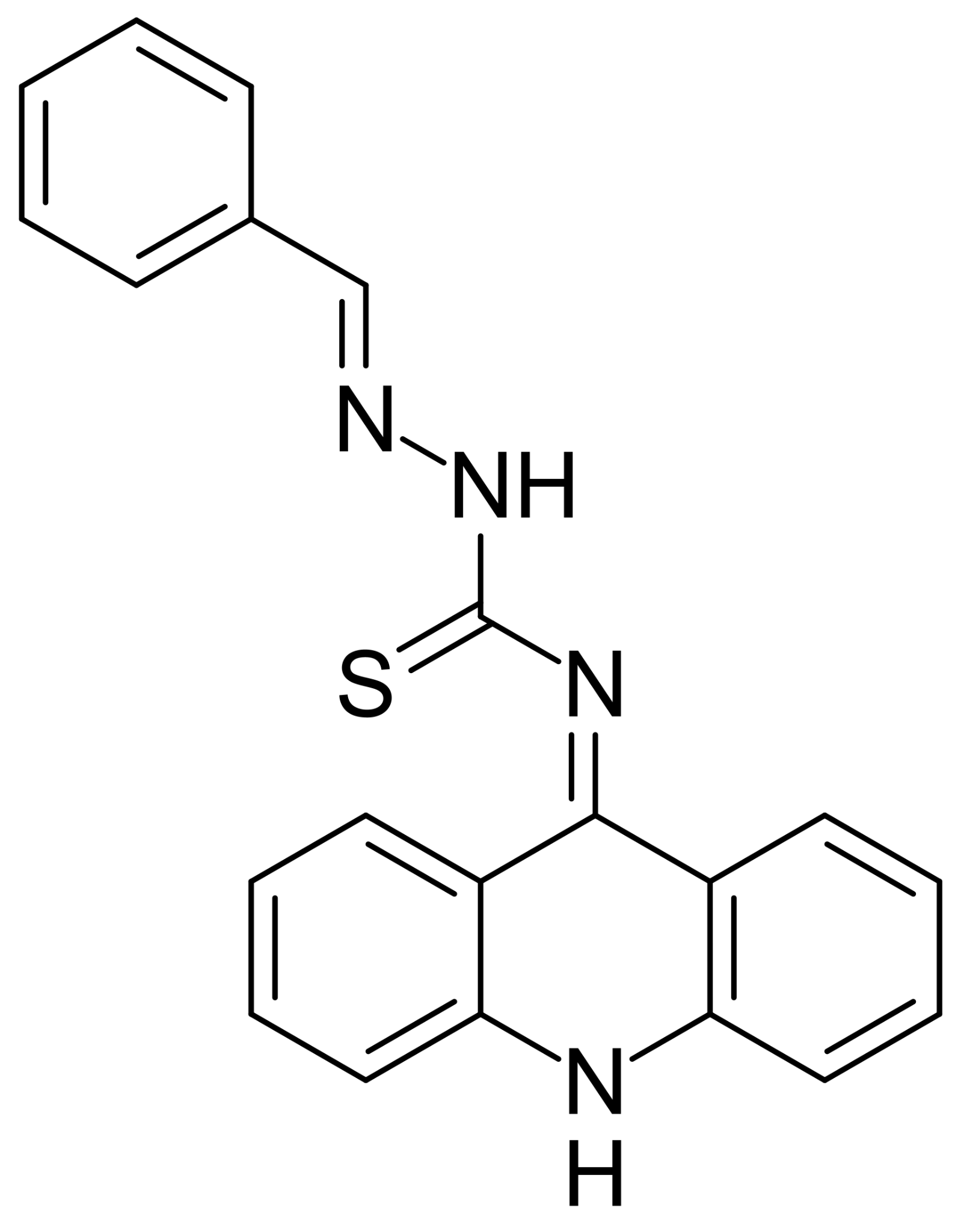 | (E)-N-(acridin-9(10H)-ylidene)-2-benzylidenehydrazinecarbothioamide | [27] |
| 17 | 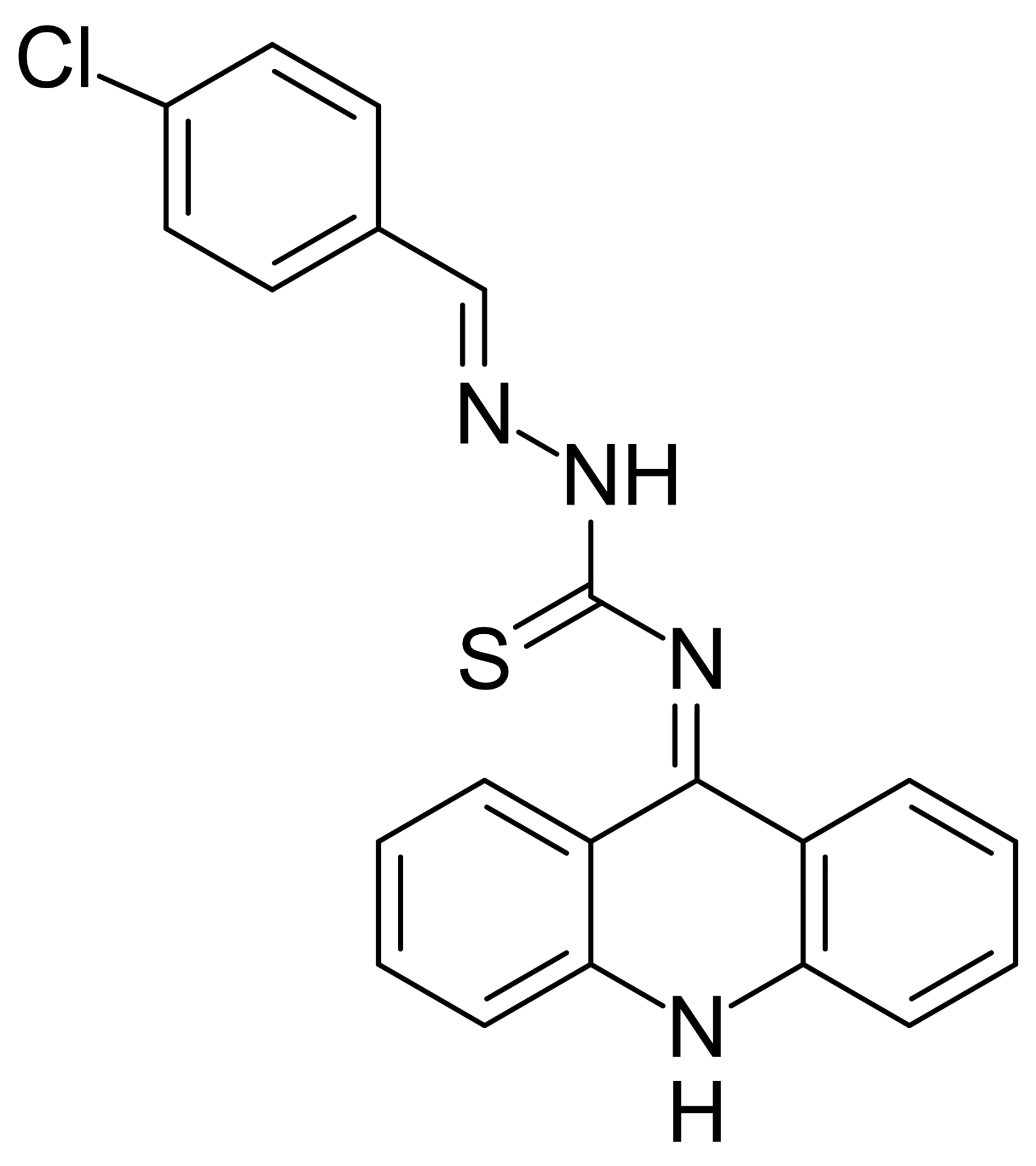 | (E)-N-(acridin-9(10H)-ylidene)-2-(4-chlorobenzylidene)hydrazinecarbothioamide | [27] |
| 18 | 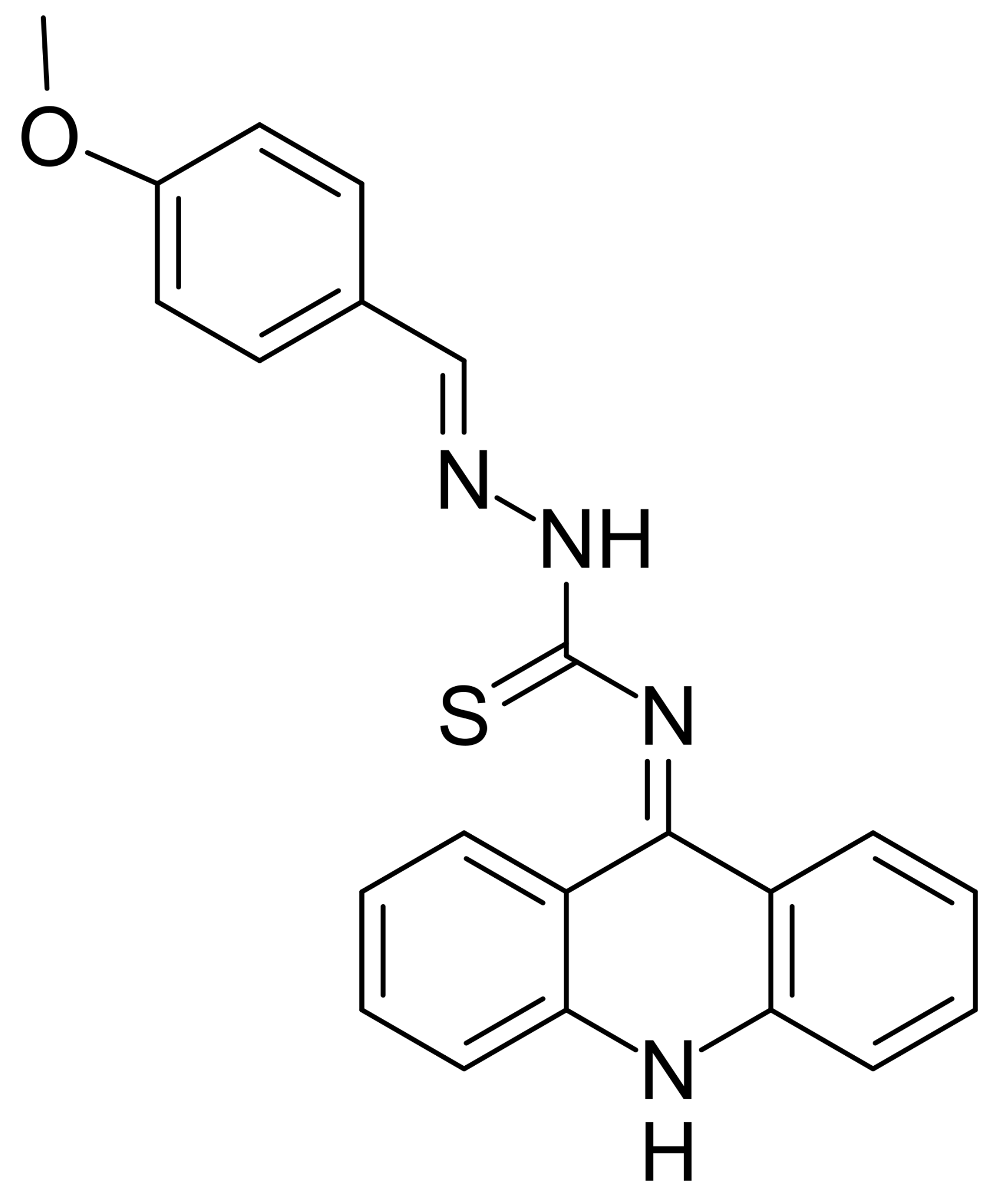 | (E)-N-(acridin-9(10H)-ylidene)-2-(4-methoxybenzylidene)hydrazinecarbothioamide | [27] |
| 19 | 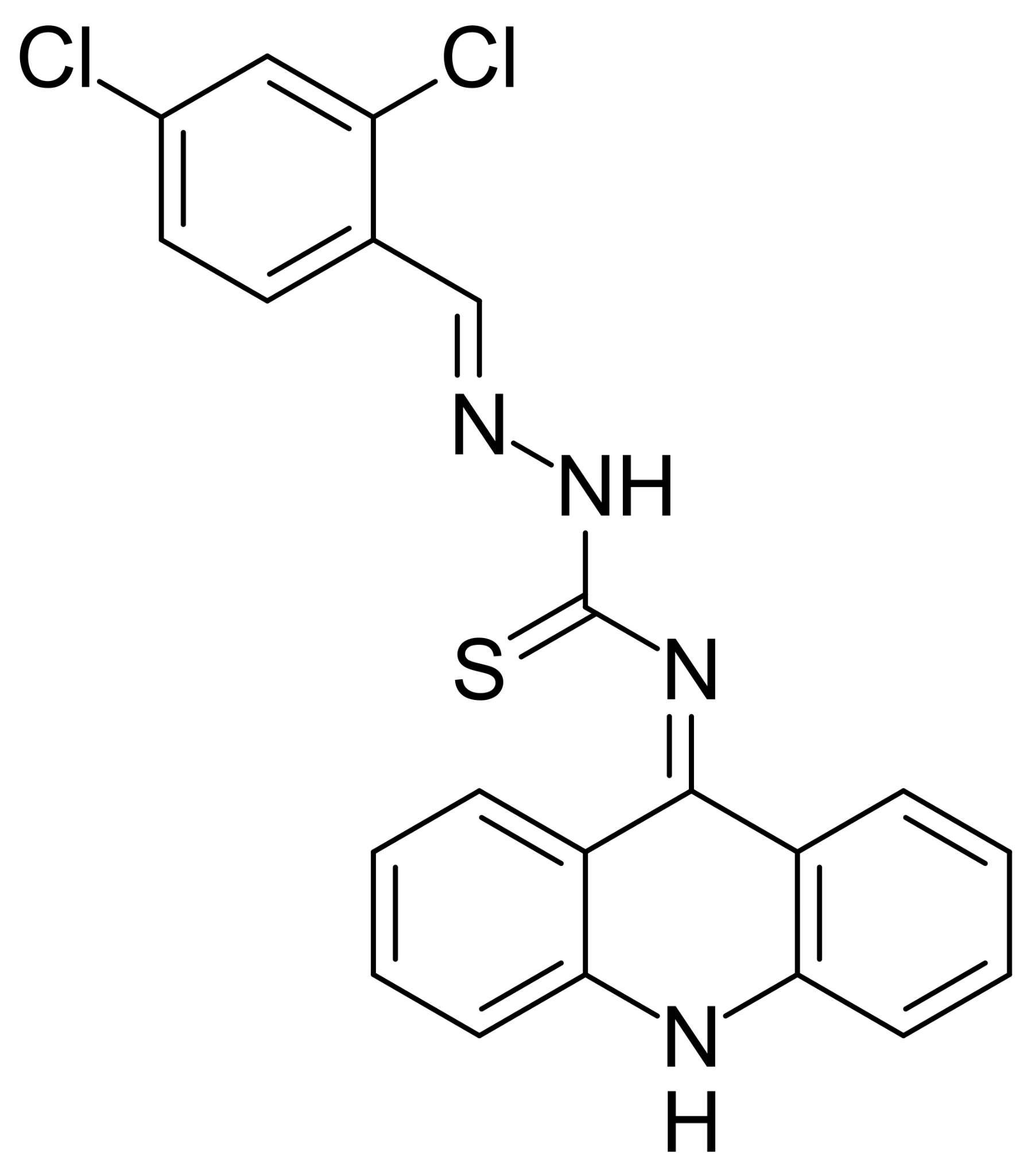 | (E)-N-(acridin-9(10H)-ylidene)-2-(2,4-dichlorobenzylidene)hydrazinecarbothioamide | [27] |
| 20 | 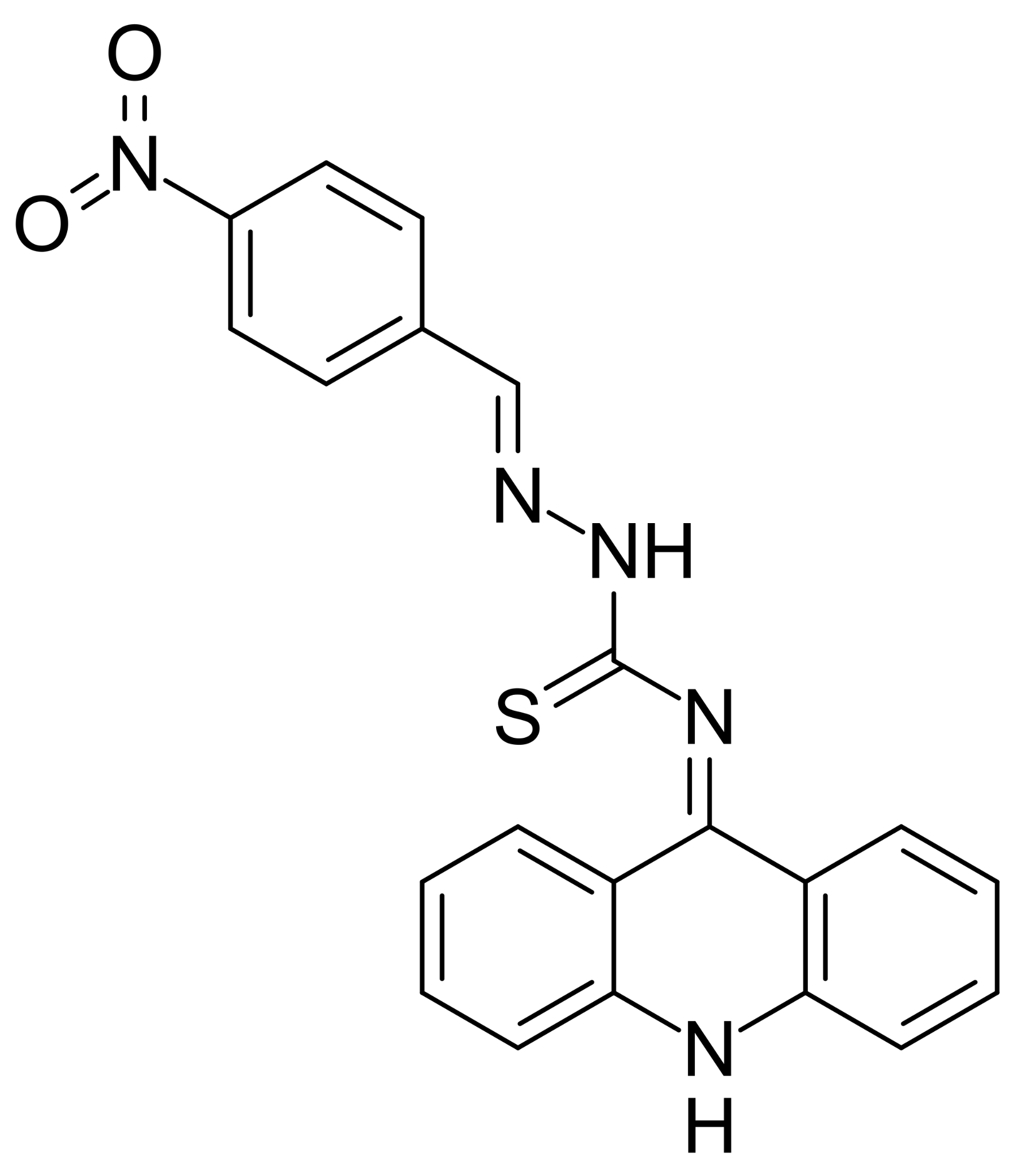 | (E)-N-(acridin-9(10H)-ylidene)-2-(4-nitrobenzylidene)hydrazinecarbothioamide | [27] |
| 21 | 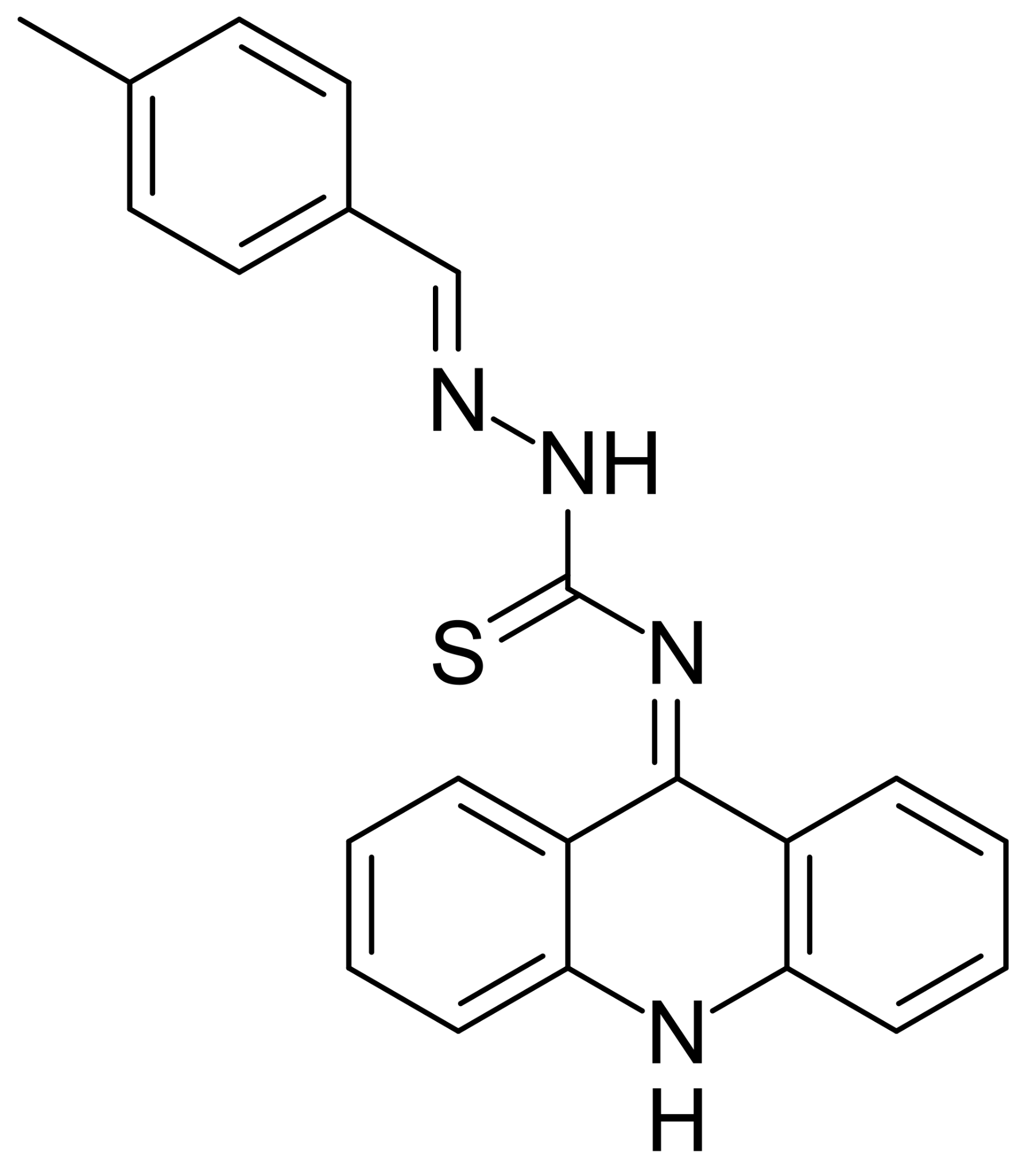 | (E)-N-(acridin-9(10H)-ylidene)-2-(4-methylbenzylidene)hydrazinecarbothioamide | [27] |
| 22 | 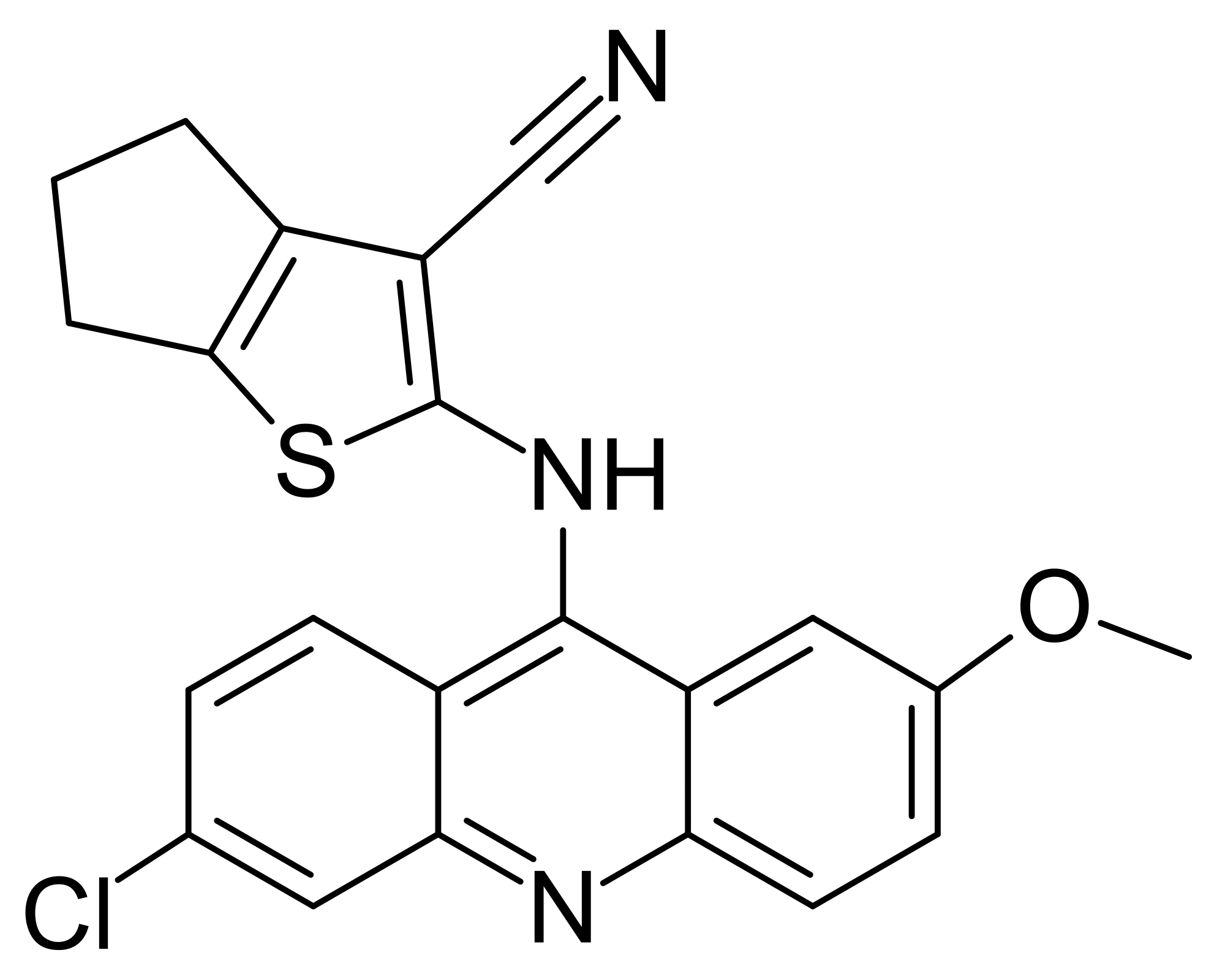 | 2-(6-chloro-2-methoxy-acridin-9-ylamino)-5,6-dihydro-4H-cyclopenta[b]- thiophene-  3-carbonitrile | [17] |
| 23 | 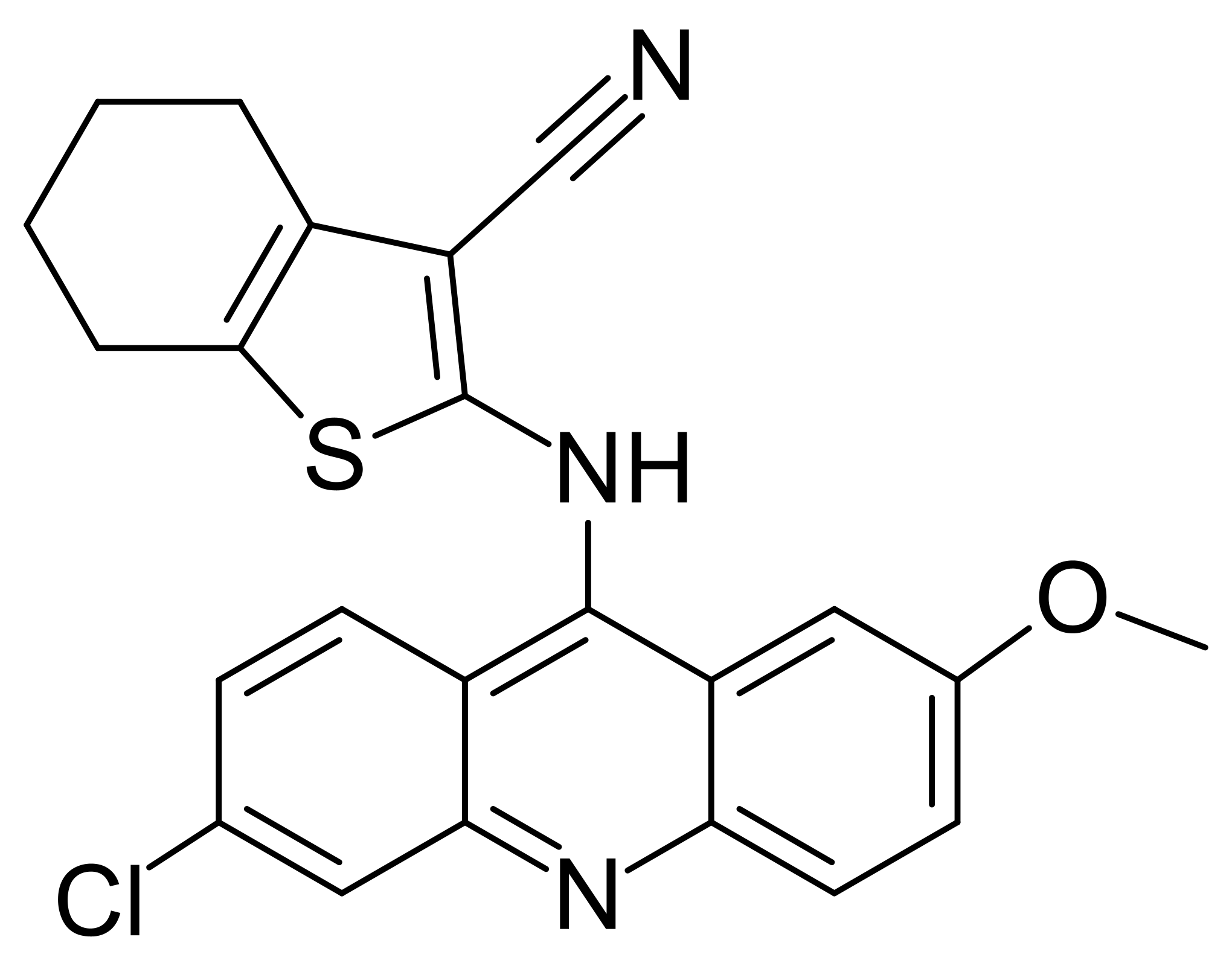 | 2-(6-Chloro-2-methoxy-acridin-9-ylamino)-4,5,6,7-tetrahydro-benzo[b]-thiophene-3-carbonitrile | [17] |
| 24 | 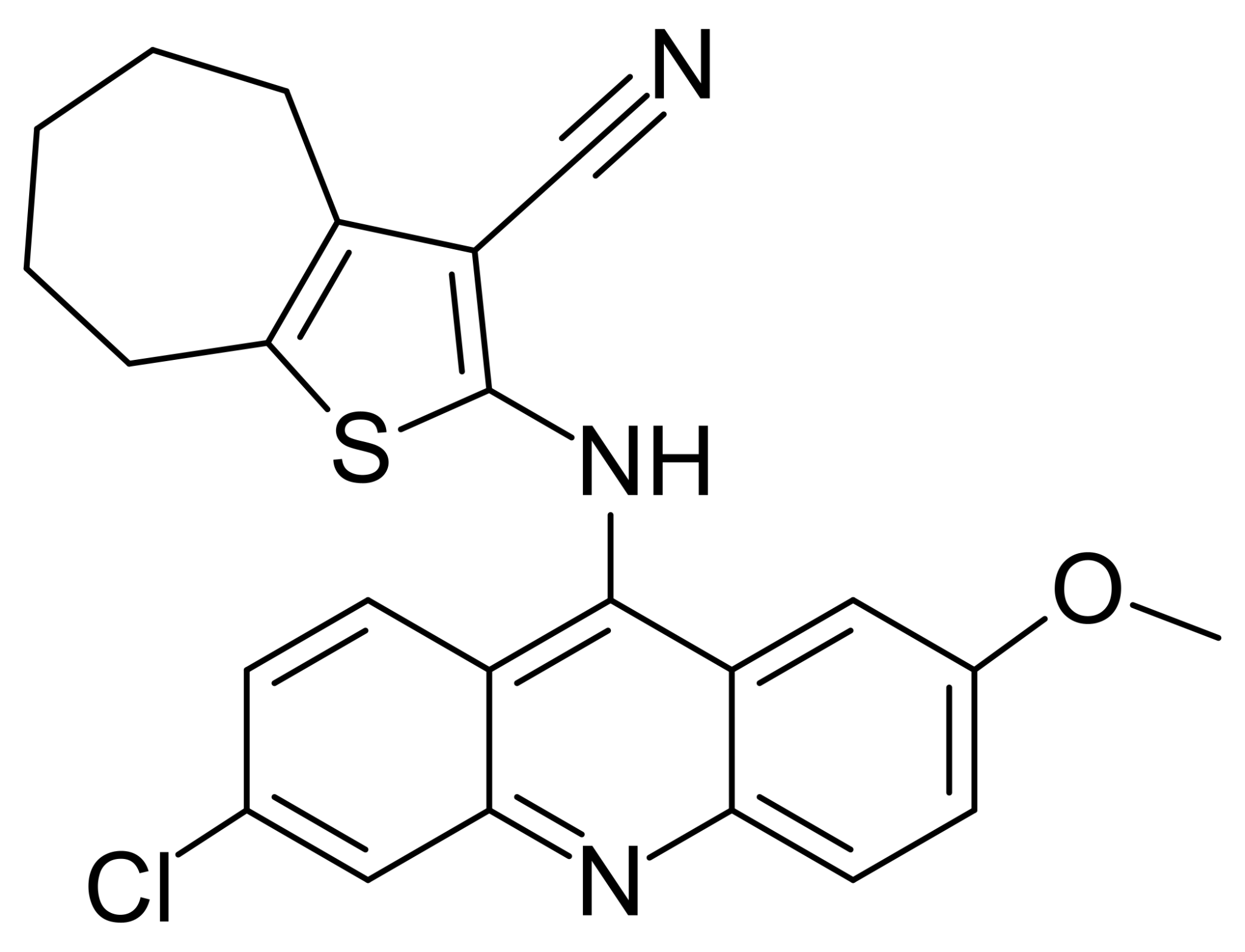 | 2-(6-Chloro-2-methoxy-acridin-9-ylamino)-5,6,7,8-tetrahydro-4H-cyclohepta[b]-thiophene-3-carbonitrile | [17] |
| 25 | 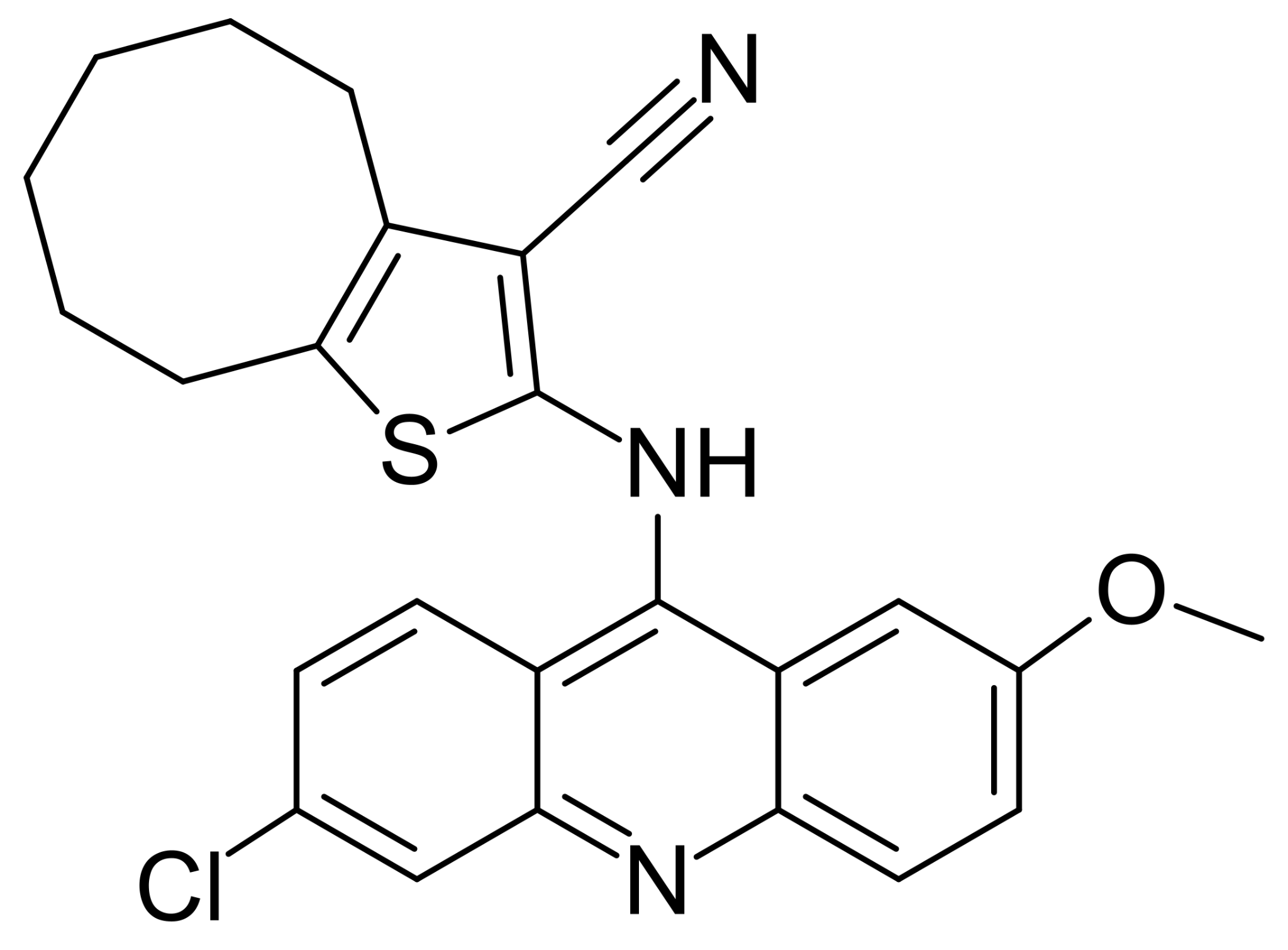 | 2-(6-Chloro-2-methoxy-acridin-9-ylamino)-4,5,6,7,8,9-hexahydro-cycloocta[b]-  thiophene-3-carbonitrile | [17] |
| 26 | 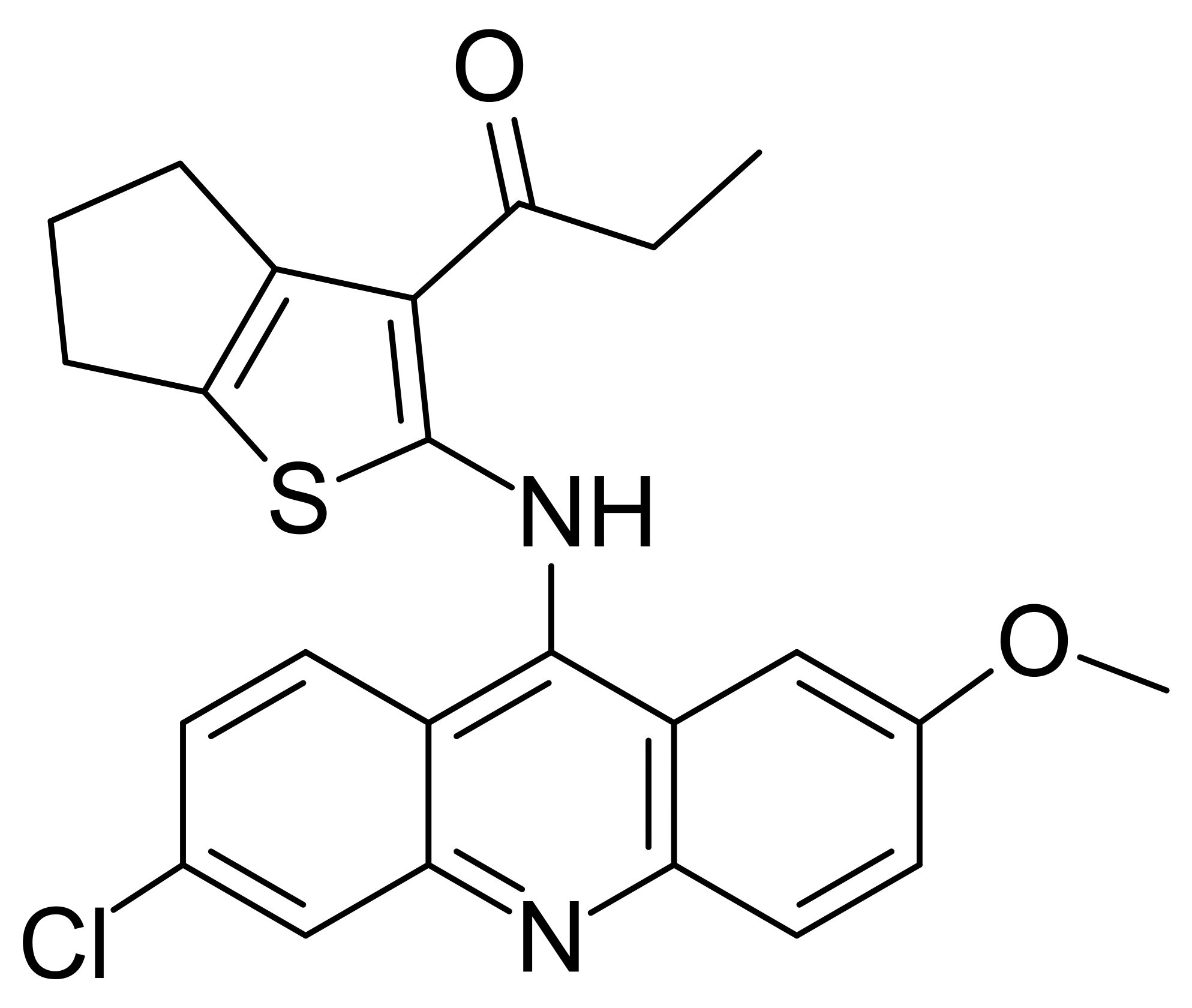 | 2-(6-Chloro-2-methoxy-acridin-9-ylamino)-4,5,6,7-tetrahydro-benzo[b]thiophene-3-  carboxylic acid ethyl ester | [17] |
| 27 | 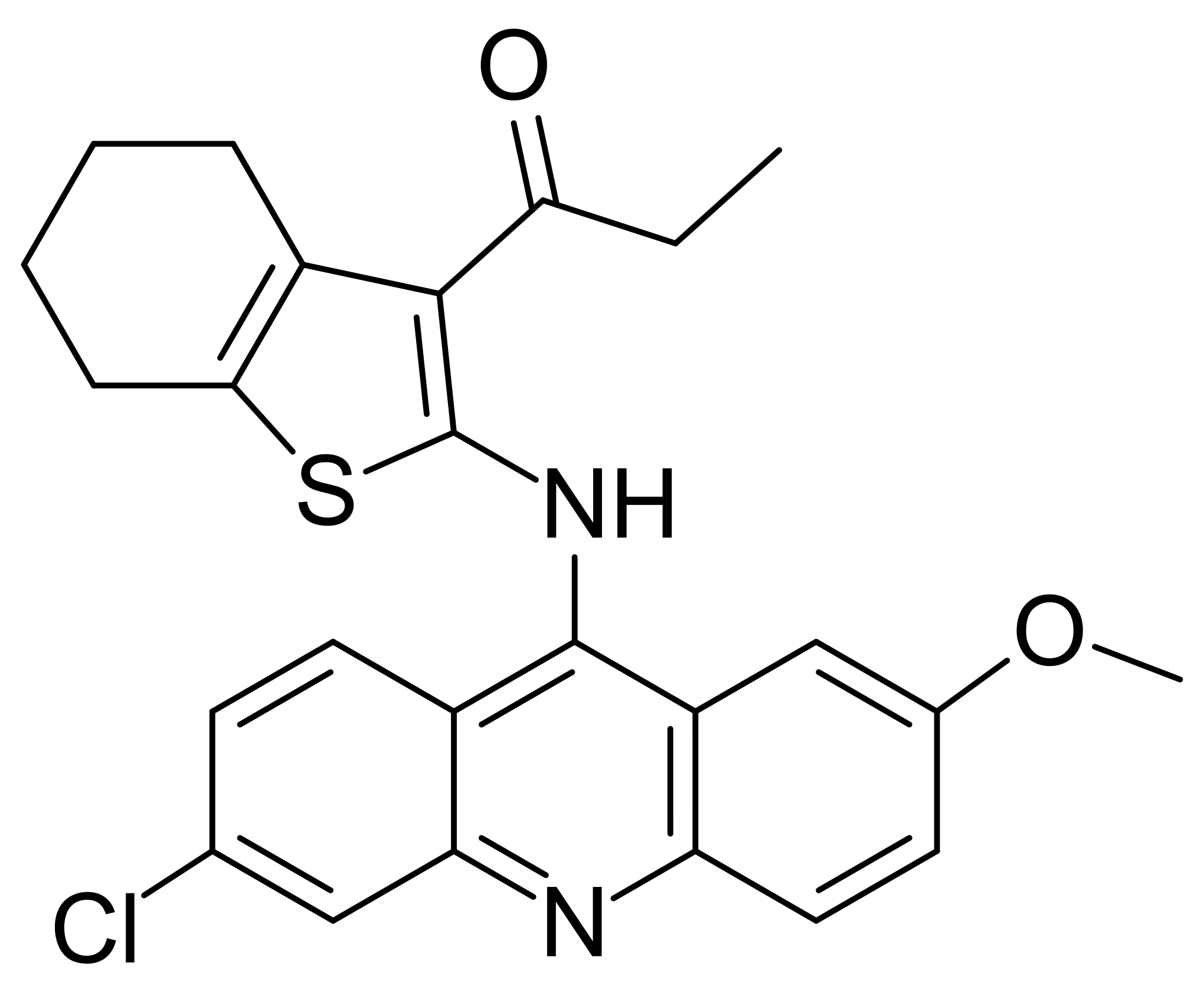 | 2-(6-Chloro-2-methoxy-acridin-9-ylamino)-5,6,7,8-tetrahydro-4H-cyclohepta[b]  thiophene-3-carboxylic acid ethyl ester | [17] |
| 28 | 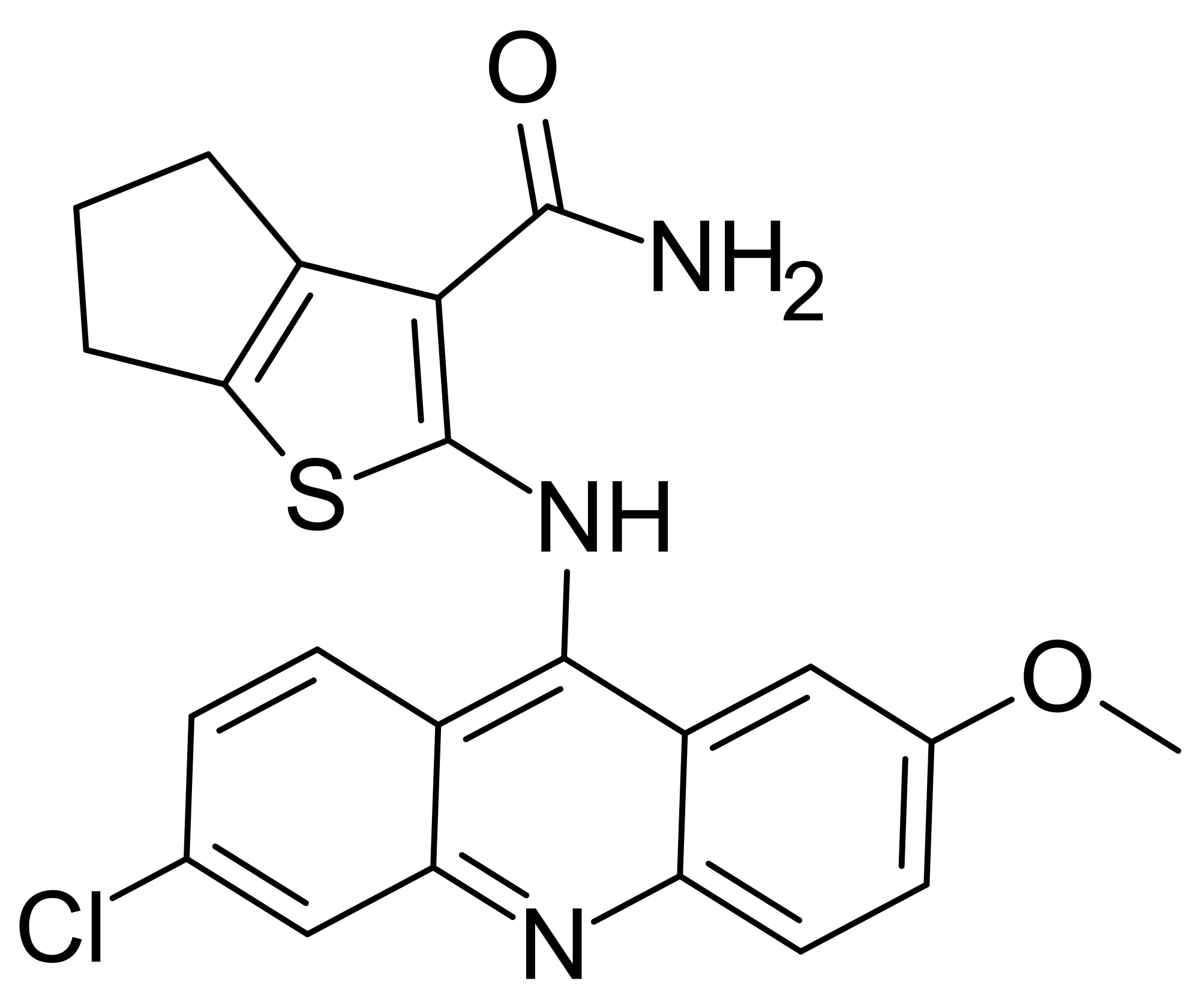 | 2-(6-Chloro-2-methoxy-acridin-9-ylamino)-4,5,6,7-tetrahydro-benzo[b]thiophene-3-  carboxylic acid amide | [17] |


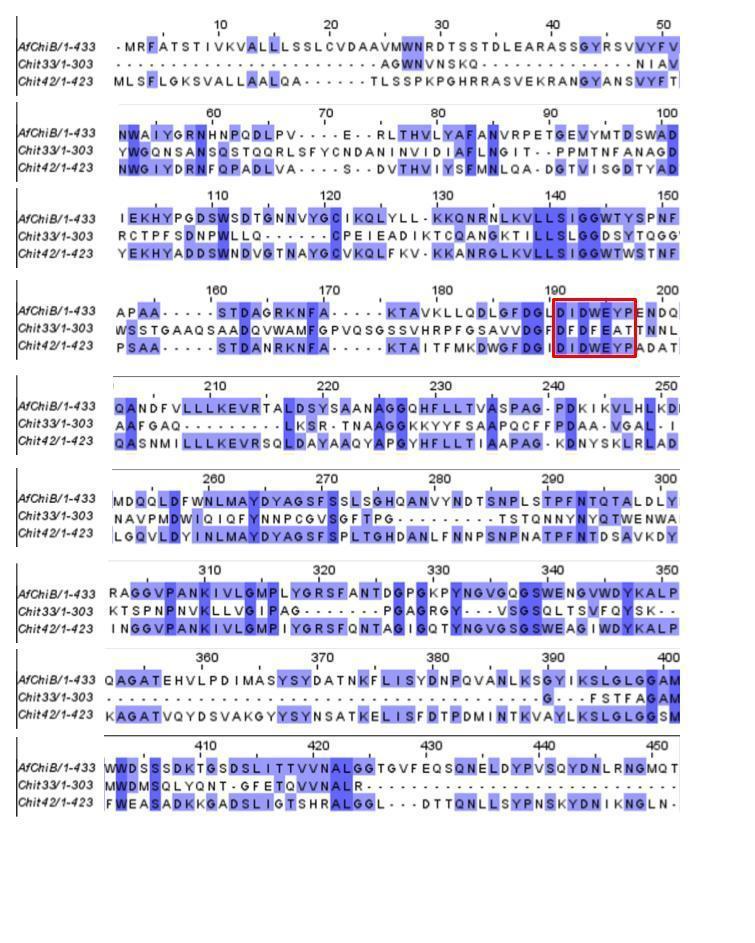


**Figure S1.** Alignment of chitinases enzymes belonging to the GH18 family of *A. fumigatus* (*Af*ChiB) and *T. harzianum* species (Chit33 and Chit42). Colored in purple, the identity of the sequences is marked. The red box is the DXDXE domain.

| 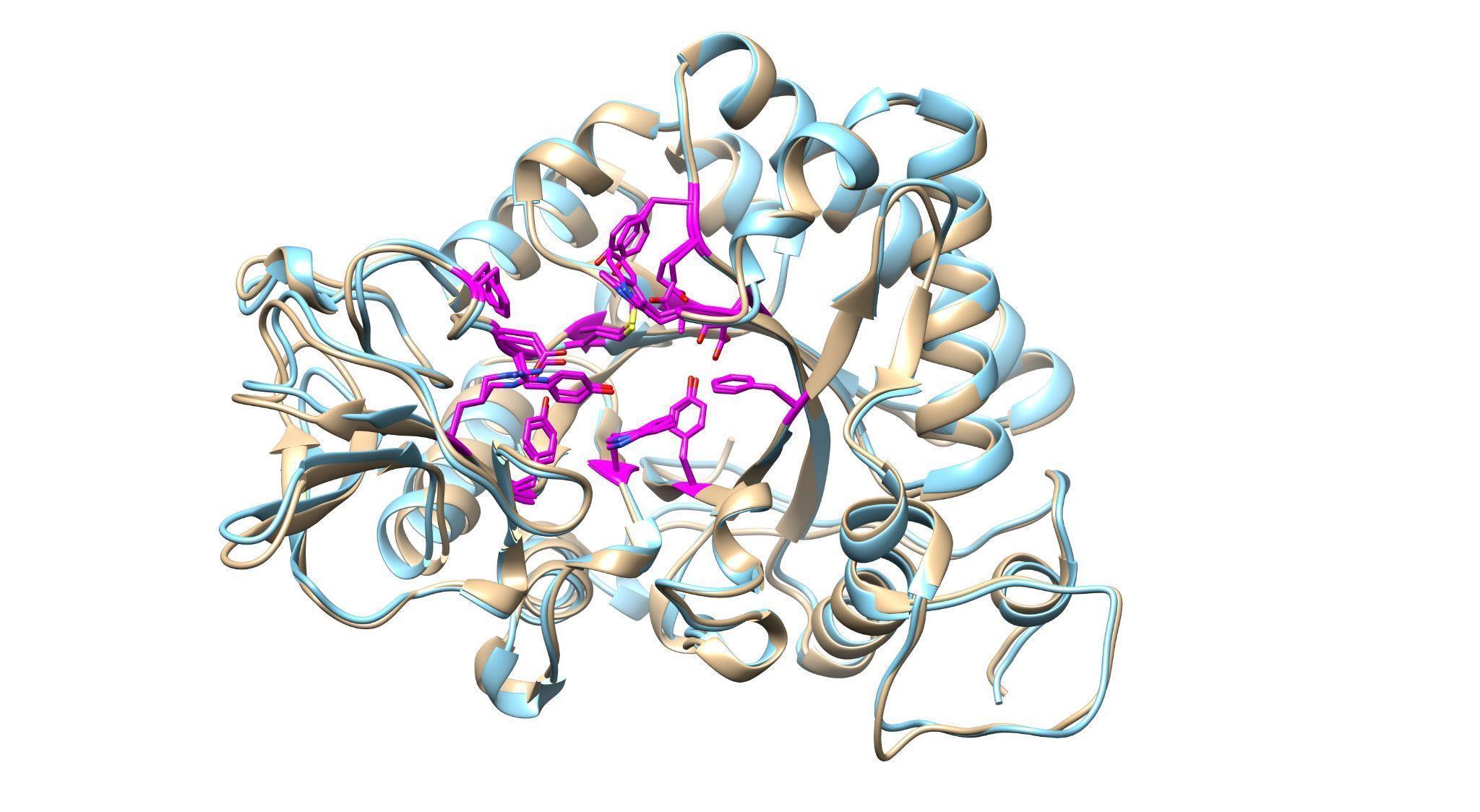 | 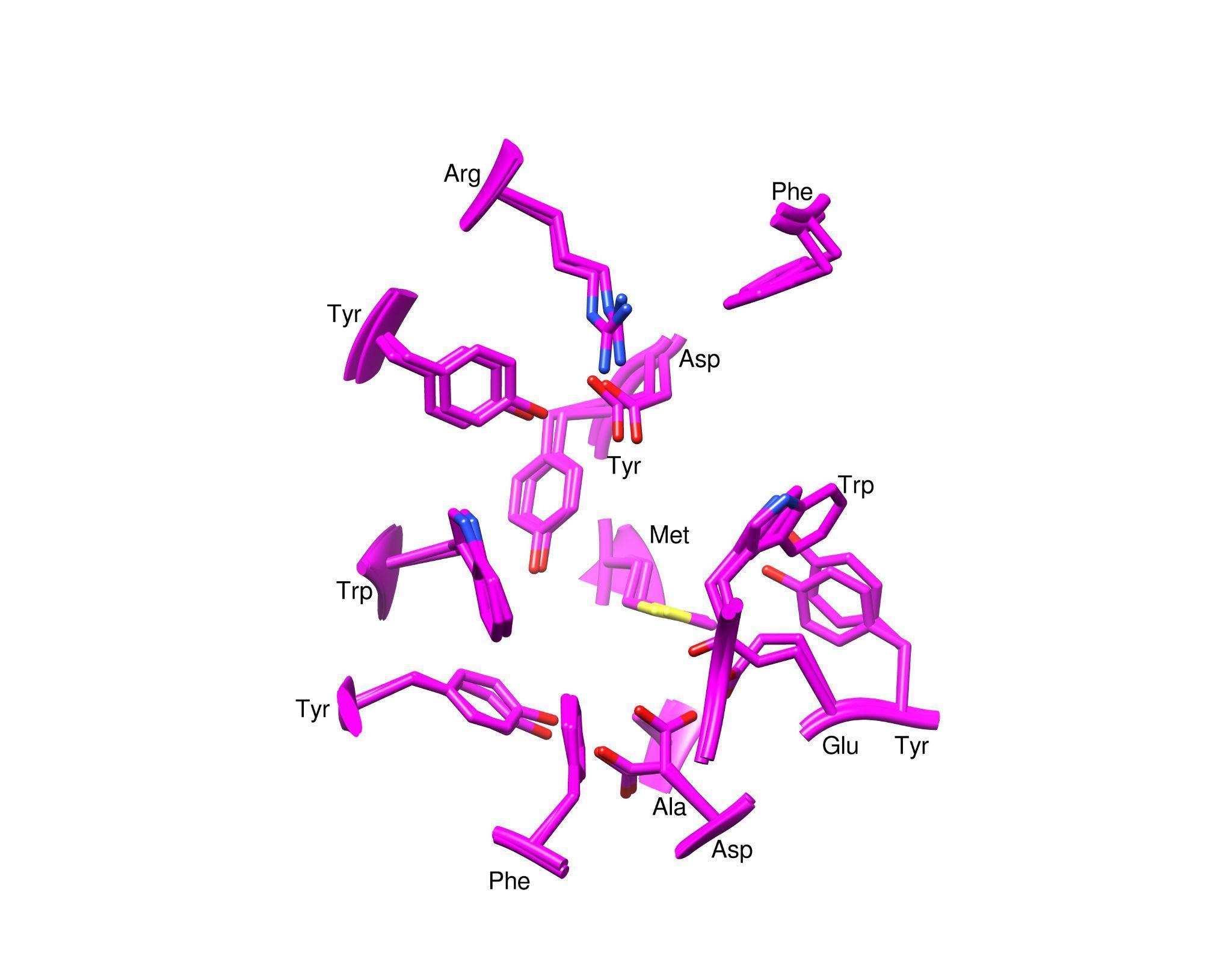 |
| --- | --- |

**Figure S2.** 3D Alignment of chitinases enzymes belonging to the GH18 family of *A. fumigatus* (*Af*ChiB, in blue color) and *T. harzianum* species (Chit42, in gold color). The active site is presented in pink color.

| 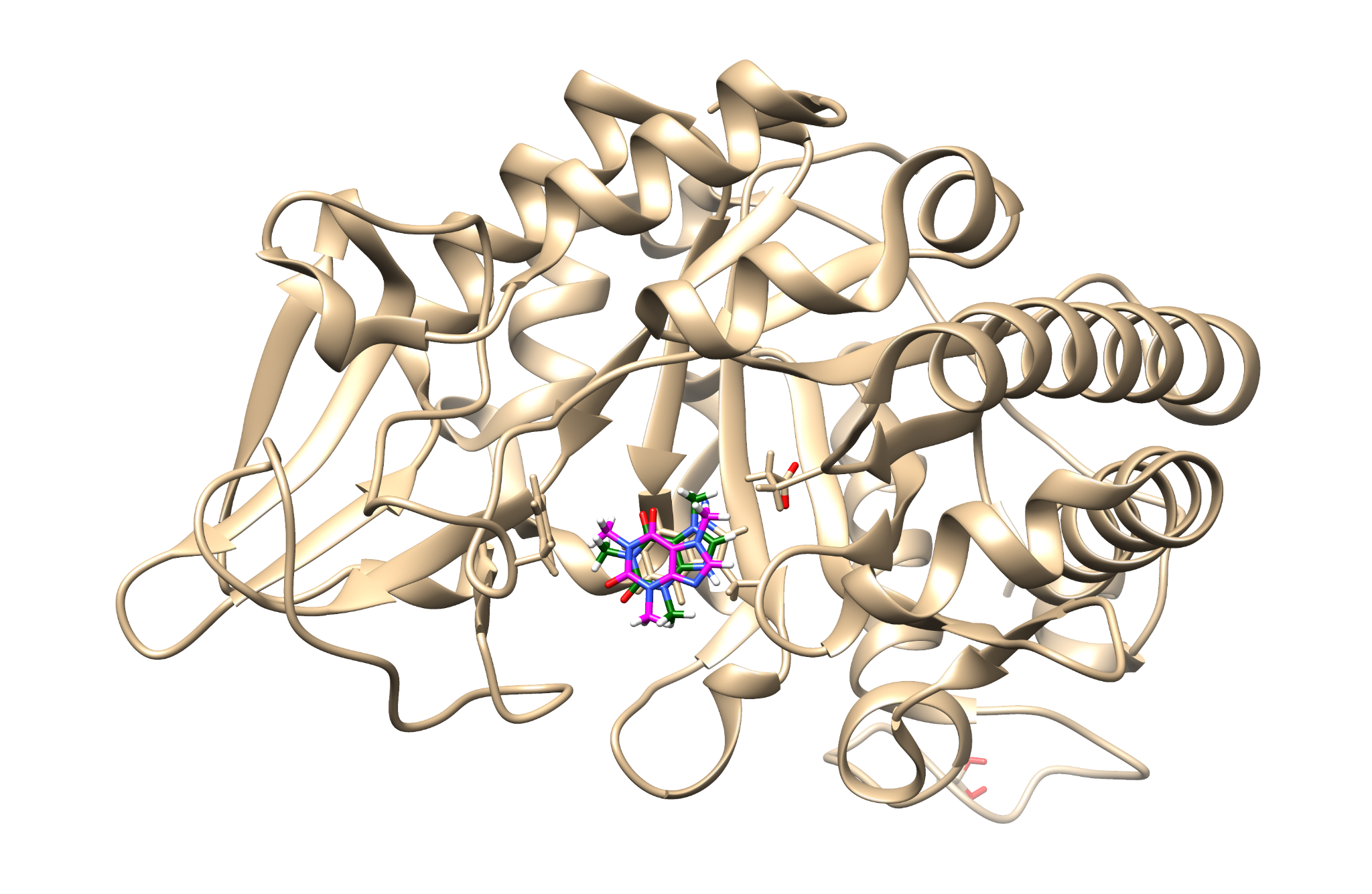 | 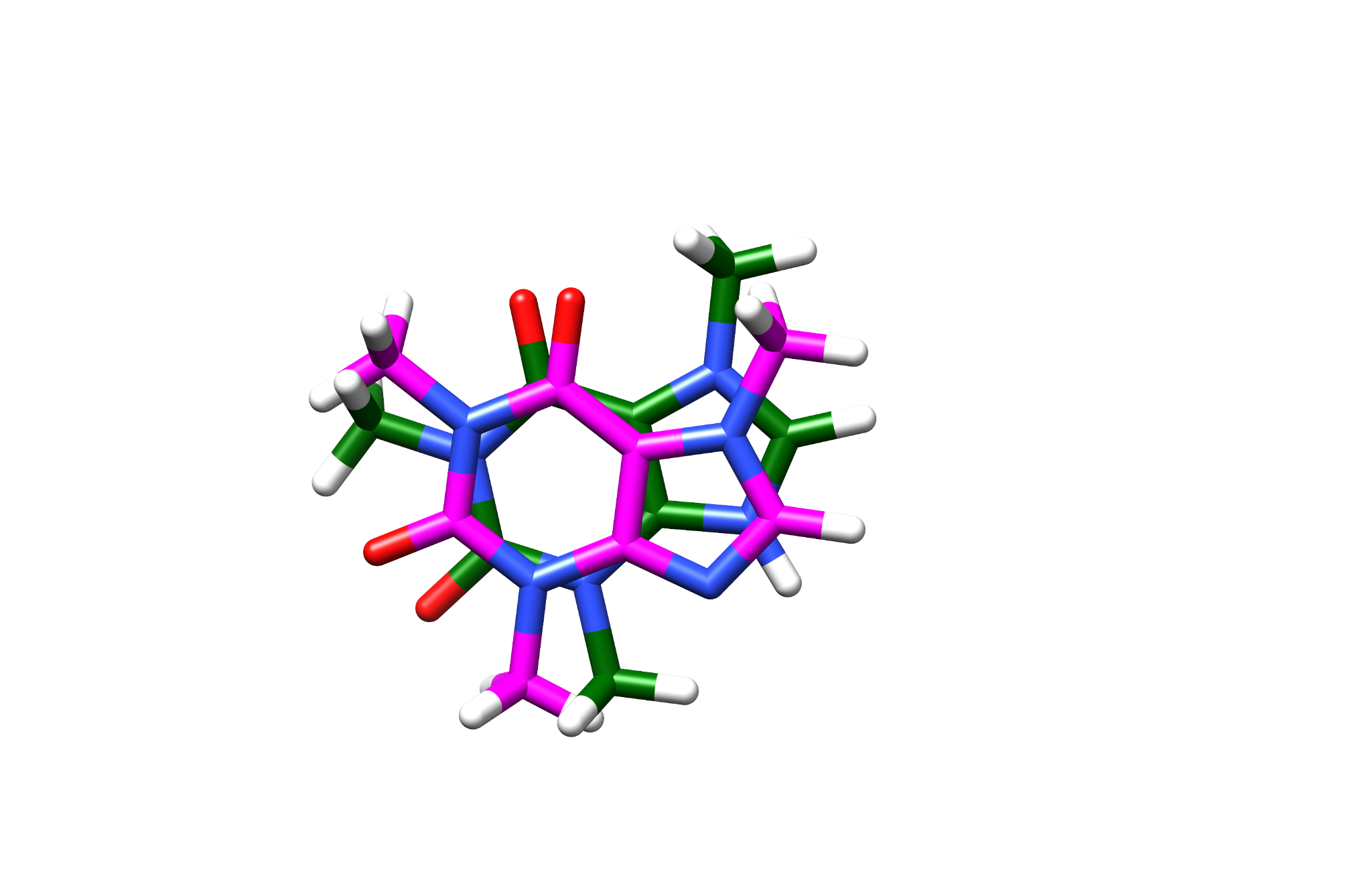 |
| --- | --- |

**Figure S3.** Redocking performed for the *A. fumigatus* chitinase inhibitor (PDB ID 2A3B). The protein is represented in a gold color. Colored pink is crystallized caffeine. The caffeine simulated is in green color. The other colors in the ligand represent the heteroatoms present in the structure: red is oxygen, blue is nitrogen and white is hydrogen.

| **A**  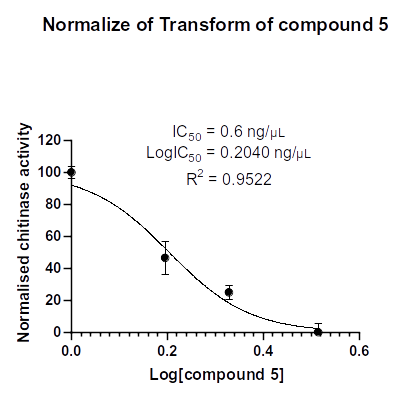 | 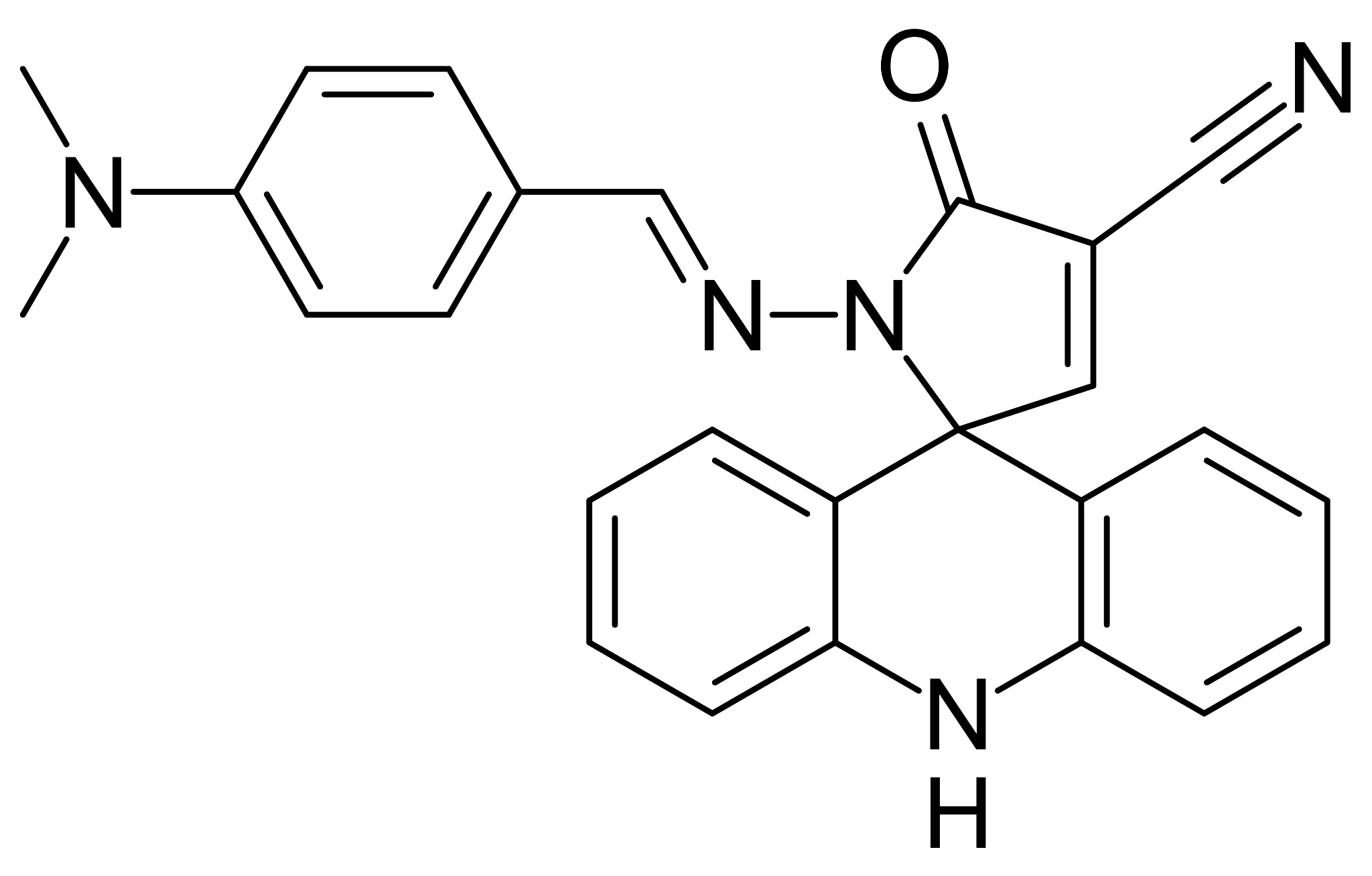 |
| --- | --- |
| **B**  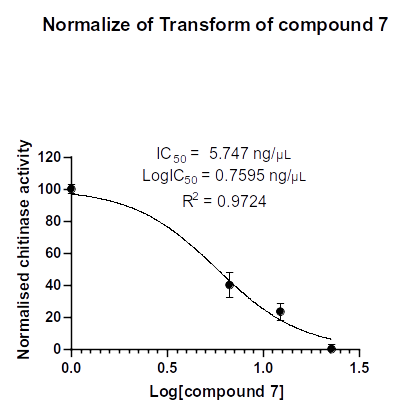 | 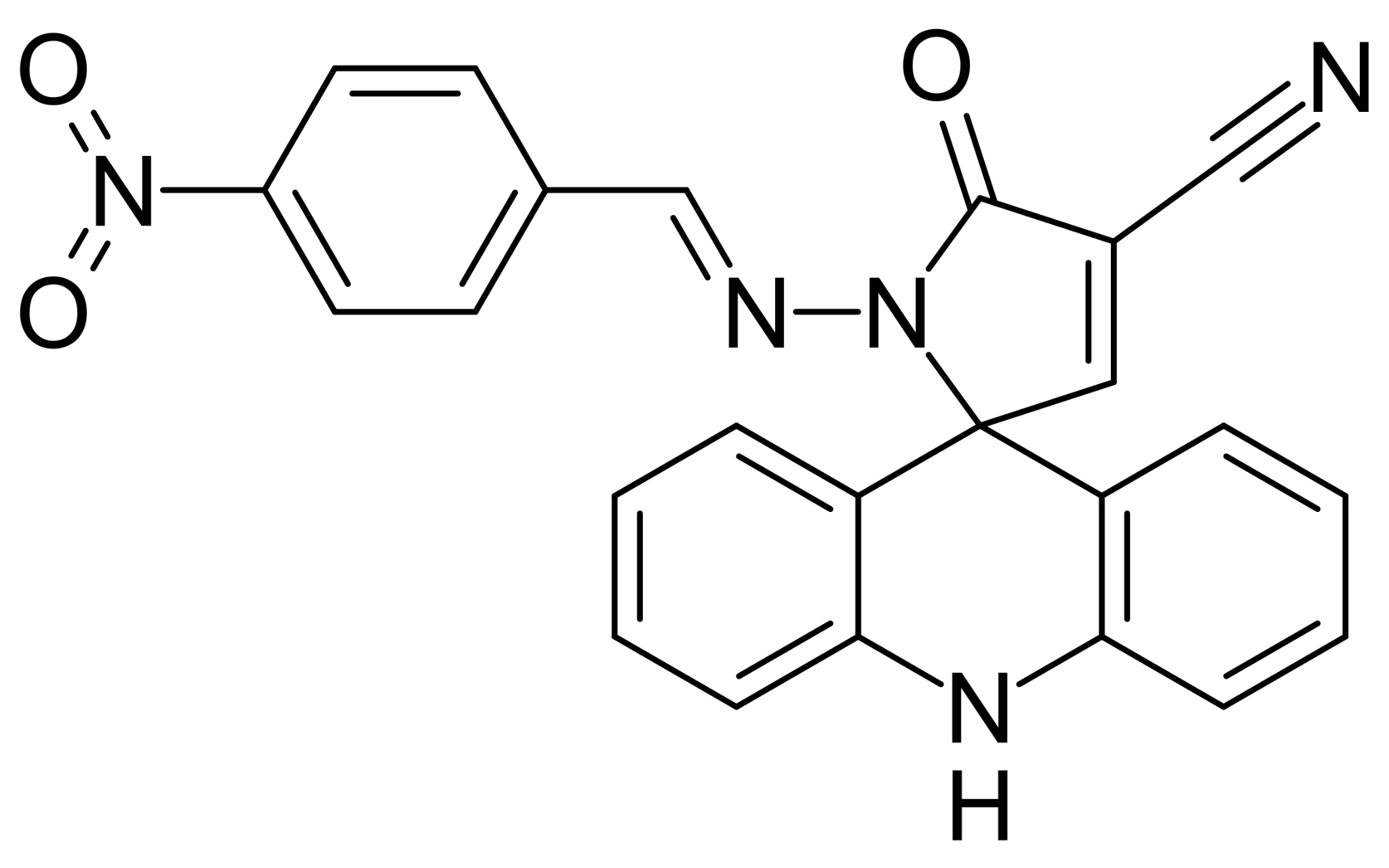 |
| **C**  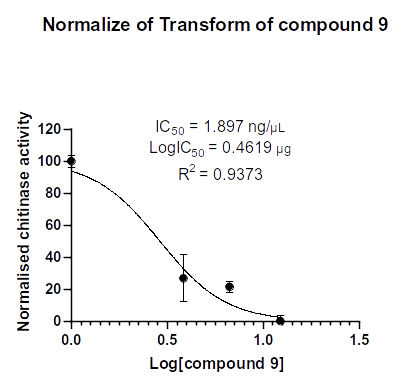 | 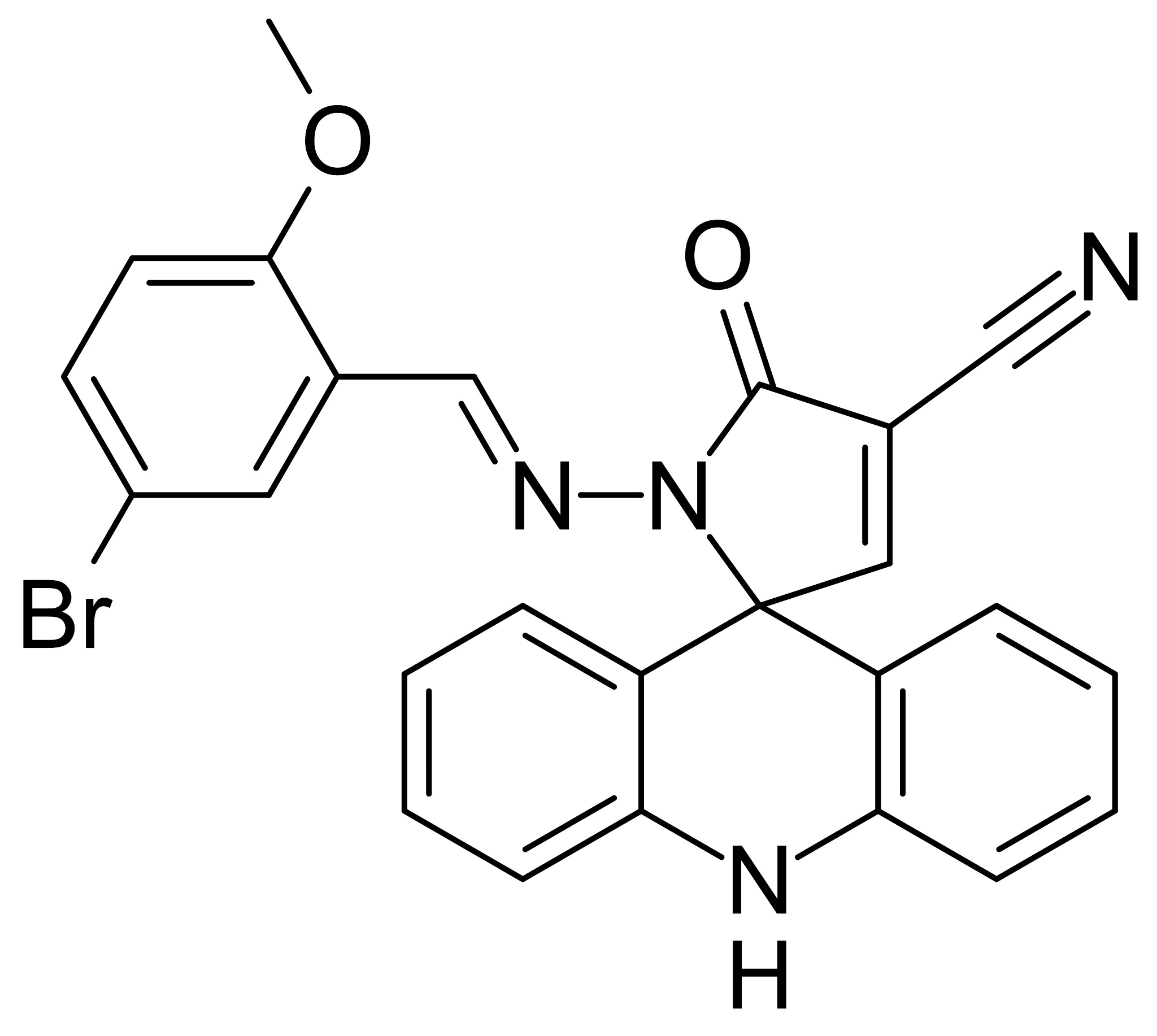 |

**Figure S4.**  Graph of IC_50_ value at 50% inhibition of three best acridine derivatives. **(A)** compound 5 **(B)** compound 7 and **(C)** compound 9.


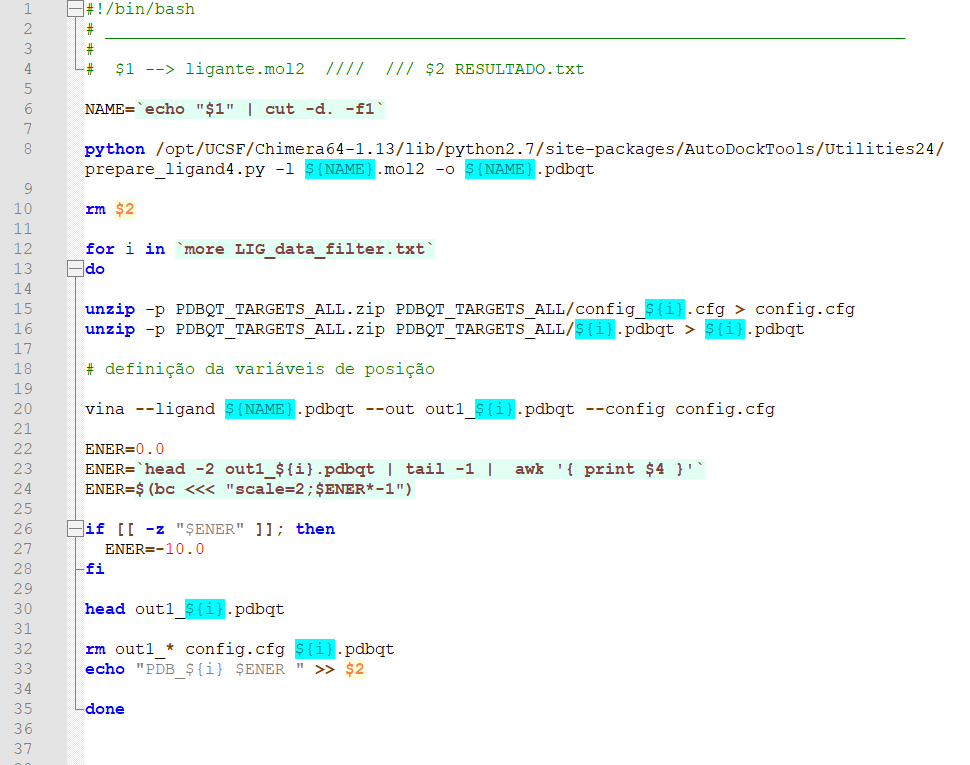


**Figure S5.**  Ad hoc script created for computing the IVS methodology.
